# Upper Palaeolithic genomes reveal deep roots of modern Eurasians

**DOI:** 10.1038/ncomms9912

**Published:** 2015-11-16

**Authors:** Eppie R. Jones, Gloria Gonzalez-Fortes, Sarah Connell, Veronika Siska, Anders Eriksson, Rui Martiniano, Russell L. McLaughlin, Marcos Gallego Llorente, Lara M. Cassidy, Cristina Gamba, Tengiz Meshveliani, Ofer Bar-Yosef, Werner Müller, Anna Belfer-Cohen, Zinovi Matskevich, Nino Jakeli, Thomas F. G. Higham, Mathias Currat, David Lordkipanidze, Michael Hofreiter, Andrea Manica, Ron Pinhasi, Daniel G. Bradley

**Affiliations:** 1 Smurfit Institute of Genetics, Trinity College Dublin, Dublin, Dublin 2, Ireland; 2 Department of Mathematics and Natural Sciences, Institute of Biochemistry and Biology, University of Potsdam, Karl-Liebknecht-Straße 24–25, Potsdam 14476, Germany; 3 Department of Biology and Evolution, University of Ferrara, Via L. Borsari 46, Ferrara I-44100, Italy; 4 School of Archaeology and Earth Institute, University College Dublin, Belfield, Dublin 4, Ireland; 5 Department of Zoology, University of Cambridge, Cambridge, CB2 3EJ, UK; 6 Integrative Systems Biology Laboratory, Division of Biological and Environmental Sciences & Engineering, King Abdullah University of Science and Technology (KAUST), Thuwal 23955-6900, Kingdom of Saudi Arabia; 7 Centre for GeoGenetics, Natural History Museum of Denmark, University of Copenhagen, Øster Voldgade 5–7, Copenhagen 1350, Denmark; 8 Georgian National Museum, 3 Rustaveli Avenue, Tbilisi 0105, Georgia; 9 Department of Anthropology, Peabody Museum, Harvard University, Cambridge, Massachusetts 02138, USA; 10 Laboratoire d'archéozoologie, Université de Neuchâtel, Neuchâtel 2000, Switzerland; 11 Office du patrimoine et de l'archéologie de Neuchâtel, Section archéologie, LATÉNIUM, Hauterive 2068, Switzerland; 12 Institute of Archaeology, Hebrew University, Jerusalem 91905, Israel; 13 Israel Antiquities Authority, PO Box 586, Jerusalem 91004, Israel; 14 Oxford Radiocarbon Accelerator Unit, Research Laboratory for Archaeology & the History of Art, University of Oxford, Oxford OX1 3QY, UK; 15 Laboratory of Anthropology, Genetics and Peopling History (AGP), Department of Genetics and Evolution - Anthropology Unit, University of Geneva, Geneva 1227, Switzerland

## Abstract

We extend the scope of European palaeogenomics by sequencing the genomes of Late Upper Palaeolithic (13,300 years old, 1.4-fold coverage) and Mesolithic (9,700 years old, 15.4-fold) males from western Georgia in the Caucasus and a Late Upper Palaeolithic (13,700 years old, 9.5-fold) male from Switzerland. While we detect Late Palaeolithic–Mesolithic genomic continuity in both regions, we find that Caucasus hunter-gatherers (CHG) belong to a distinct ancient clade that split from western hunter-gatherers ∼45 kya, shortly after the expansion of anatomically modern humans into Europe and from the ancestors of Neolithic farmers ∼25 kya, around the Last Glacial Maximum. CHG genomes significantly contributed to the Yamnaya steppe herders who migrated into Europe ∼3,000 BC, supporting a formative Caucasus influence on this important Early Bronze age culture. CHG left their imprint on modern populations from the Caucasus and also central and south Asia possibly marking the arrival of Indo-Aryan languages.

## Abstract (web summary)

Upper Palaeolithic and Mesolithic genomes from western Europe and the Caucasus reveal a previously undescribed strand of Eurasian ancestry with a deep divergence from other hunter-gatherer genomes. This had a profound impact on ancient and modern populations from the Atlantic to Central Asia.

## Graphical abstract

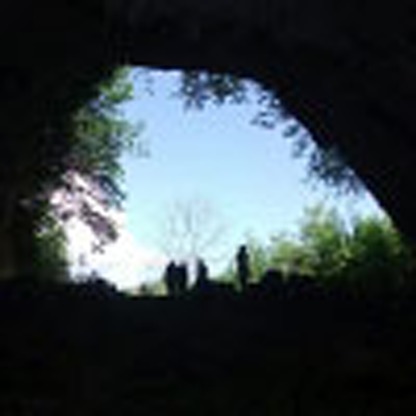

Ancient genomes from Eurasia have revealed three ancestral populations that contributed to contemporary Europeans in varying degrees^1^. Mesolithic individuals, sampled from Spain all the way to Hungary^1^,^2^,^3^, belong to a relatively homogenous group, termed western hunter-gatherers (WHG). The expansion of early farmers (EF) out of the Levant during the Neolithic transition led to major changes in the European gene pool, with almost complete replacement in the south and increased mixing with local WHG further north^1^,^2^,^3^,^4^,^5^. Finally, a later wave originating with the Early Bronze Age Yamnaya from the Pontic steppe, carrying partial ancestry from ancient North Eurasians (ANE) and ancestry from a second, undetermined source, arrived from the east, profoundly changing populations and leaving a cline of admixture in Eastern and Central Europe^1^,^3^,^6^. This view, which was initially based on a handful of genomes, was recently confirmed by extensive surveys of Eurasian samples from the Holocene^5^,^7^.

Here, we extend our view of the genetic makeup of early Europeans by both looking further back in time and sampling from the crossroads between the European and Asian continents. We sequenced a Late Upper Palaeolithic (‘Satsurblia' from Satsurblia cave, 1.4-fold coverage) and a Mesolithic genome (‘Kotias' from Kotias Klde cave, 15.4-fold) from Western Georgia, at the very eastern boundary of Europe. We term these two individuals Caucasus hunter-gatherers (CHG). To extend our overview of WHG to a time depth similar to the one available for our samples from the Caucasus, we also sequenced a western European Late Upper Palaeolithic genome, ‘Bichon' (9.5-fold) from Grotte du Bichon, Switzerland. These new genomes, together with already published data, provide us with a much-improved geographic and temporal coverage of genetic diversity across Europe after the Last Glacial Maximum (LGM)^8^. We show that CHG belong to a new, distinct ancient clade that split from WHG ∼45 kya and from Neolithic farmer ancestors ∼25 kya. This clade represents the previously undetermined source of ancestry to the Yamnaya, and contributed directly to modern populations from the Caucasus all the way to Central Asia.

## Results

### Samples, sequencing and authenticity

Recent excavations of Satsurblia cave in Western Georgia yielded a human right temporal bone, dated to the Late Upper Palaeolithic 13,132–13,380 cal. BP. Following the approach of Gamba *et al.*^3^, extractions from the dense part of the petrous bone yielded sequencing libraries comprising 13.8% alignable human sequence which were used to generate 1.4-fold genome coverage. A molar tooth sampled from a later Mesolithic (9,529–9,895 cal. BP) burial in Kotias Klde, a rockshelter also in Western Georgia showed excellent preservation, with endogenous human DNA content of 76.9%. This was sequenced to 15.4-fold genome coverage. Grotte du Bichon is a cave situated in the Swiss Jura Mountains where a skeleton of a young male of Cro-magnon type was found and dated to the late Upper Palaeolithic 13,560–13,770 cal. BP (for further details on the archaeological context see Supplementary Note 1). A petrous bone sample extraction from this also gave excellent endogenous content at 71.5% and was sequenced to 9.5-fold coverage. The sequence data from each genome showed sequence length and nucleotide misincorporation patterns which were indicative of post-mortem damage and contamination estimates, based on X chromosome and mitochondrial DNA tests (Supplementary Note 2), were <1%, comparable to those found in other ancient genomes^2^,^3^,^8^.

### Continuity across the Palaeolithic–Mesolithic boundary

**Figure 1:**
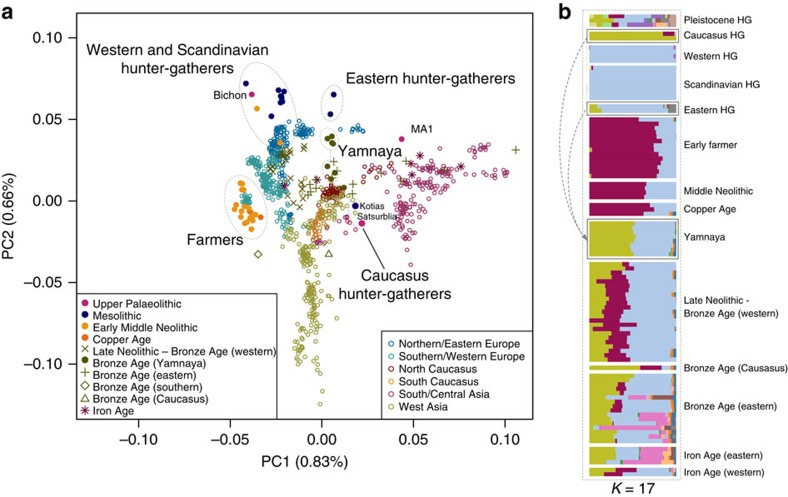
Genetic structure of ancient Europe. (**a**). Principal component analysis. Ancient data from Bichon, Kotias and Satsurblia genomes were projected^11^ onto the first two principal components defined by selected Eurasians from the Human Origins data set^1^. The percentage of variance explained by each component accompanies the titles of the axes. For context we included data from published Eurasian ancient genomes sampled from the Late Pleistocene and Holocene where at least 200 000 SNPs were called^1^,^2^,^3^,^4^,^5^,^6^,^7^,^9^ (Supplementary Table 1). Among ancients, the early farmer and western hunter-gatherer (including Bichon) clusters are clearly identifiable, and the influence of ancient north Eurasians is discernible in the separation of eastern hunter-gatherers and the Upper Palaeolithic Siberian sample MA1. The two Caucasus hunter-gatherers occupy a distinct region of the plot suggesting a Eurasian lineage distinct from previously described ancestral components. The Yamnaya are located in an intermediate position between CHG and EHG. (**b**). ADMIXTURE ancestry components^12^ for ancient genomes (*K*=17) showing a CHG component (Kotias, Satsurblia) which also segregates in in the Yamnaya and later European populations.

Kotias and Satsurblia, the two CHG, are genetically different from all other early Holocene (that is, Mesolithic and Neolithic) ancient genomes^1^,^2^,^3^,^4^,^5^,^6^,^8^,^9^,^10^, while Bichon is similar to other younger WHG. The distinctness of CHG can be clearly seen on a principal component analysis (PCA) plot^11^ loaded on contemporary Eurasian populations^1^, where they fall between modern Caucasian and South Central Asian populations in a region of the graph separated from both other hunter gatherer and EF samples (Fig. 1a). Clustering using ADMIXTURE software^12^ confirms this view, with CHG forming their own homogenous cluster (Fig. 1b). The close genetic proximity between Satsurblia and Kotias is also formally supported by *D*-statistics^13^, indicating the two CHG genomes form a clade to the exclusion of other pre-Bronze Age ancient genomes (Supplementary Table 2; Supplementary Note 3), suggesting continuity across the Late Upper Palaeolithic and Mesolithic periods. This result is mirrored in western Europe as Bichon is close to other WHG in PCA space (Fig. 1a) and outgroup *f*_3_ analysis (Supplementary Fig. 1), belongs to the same cluster as other WHG in ADMIXTURE analysis (Fig. 1b), and forms a clade with other WHG to the exclusion of other ancient genomes based on *D*-statistics (Supplementary Table 3; Supplementary Note 3). Thus, these new data indicate genomic persistence between the Late Upper Palaeolithic and Mesolithic both within western Europe and, separately, within the Caucasus.

### Deep coalescence of early Holocene lineages

**Figure 2:**
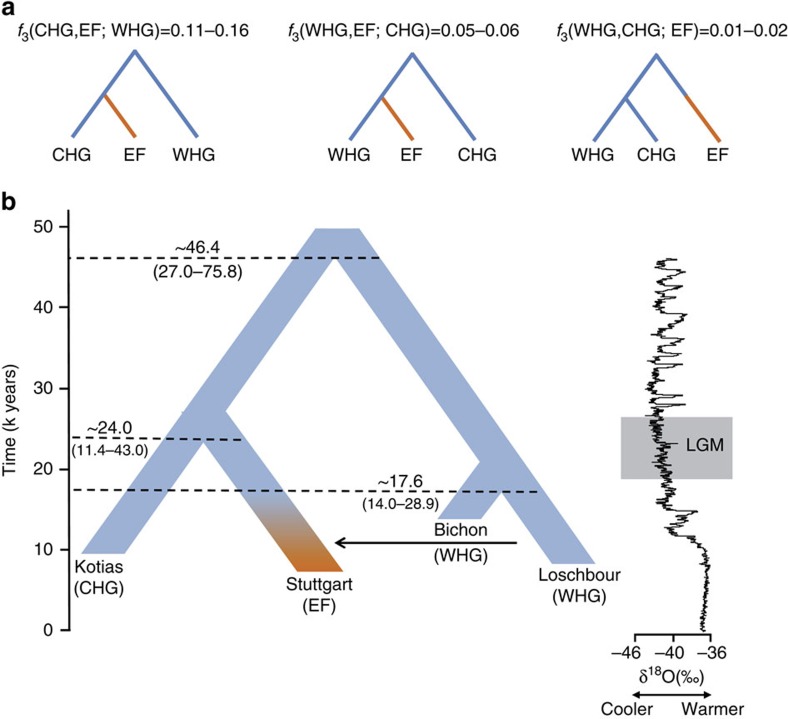
The relationship between Caucasus hunter-gatherers (CHG), western hunter-gatherers and early farmers. (**a**). Alternative phylogenies relating western hunter-gatherers (WHG), CHG and early farmers (EF, highlighted in orange), with the appropriate outgroup *f*_3_-statistics. (**b**). The best supported relationship among CHG (Kotias), WHG (Bichon, Loschbour), and EF (Stuttgart), with split times estimates using G-Phocs^15^. Oxygen 18 values (per mile) from the NGRIP core provide the climatic context; the grey box shows the extent of the Last Glacial Maximum (LGM).

The geographical proximity of the Southern Caucasus to the Levant begs the question of whether CHG might be related to early Neolithic farmers with Near Eastern heritage. To address this question formally we reconstructed the relationship among WHG, CHG and EF using available high-quality ancient genomes^1^,^3^. We used outgroup *f*_3_-statistics^14^ to compare the three possible topologies, with the correct relationship being characterized by the largest amount of shared drift between the two groups that form a clade with respect to the outgroup (Fig. 2a; Supplementary Note 4). A scenario in which the population ancestral to both CHG and EF split from WHG receives the highest support, implying that CHG and EF form a clade with respect to WHG. We can reject a scenario in which CHG and WHG form a distinct clade with respect to EF. The known admixture of WHG with EF^1^,^3^,^4^,^5^ implies that some shared drift is found between WHG and EF with respect to CHG, but this is much smaller than the shared drift between CHG and EF. Thus, WHG split first, with CHG and EF separating only at a later stage.

We next dated the splits among WHG, CHG and EF using a coalescent model implemented with G-PhoCS^15^ based on the high-coverage genomes in our data set (Fig. 2b for a model using the German farmer Stuttgart^1^ to represent EF; and Supplementary Table 5 for models using the Hungarian farmer NE1 (ref. 3)) and taking advantage of the mutation rate recently derived from Ust'-Ishim^10^. G-Phocs dates the split between WHG and the population ancestral to CHG and EF at ∼40–50 kya (range of best estimates depending on which genomes are used; see Supplementary Table 5 and Supplementary Note 5 for details), implying that they diverged early on during the colonisation of Europe^16^, and well before the LGM. On the WHG branch, the split between Bichon and Loschbour^1^ is dated to ∼16–18 kya (just older than the age of Bichon), implying continuity in western Europe, which supports the conclusions from our previous analyses. The split between CHG and EF is dated at ∼20–30 kya emerging from a common basal Eurasian lineage^1^ (Supplementary Fig. 2) and suggesting a possible link with the LGM, although the broad confidence intervals require some caution with this interpretation. In any case, the sharp genomic distinctions between these post-LGM populations contrasts with the comparative lack of differentiation between the earlier Eurasian genomes, for example, as visualized in the ADMIXTURE analysis (Fig. 1a), and it seems likely that this structure emerged as a result of ice age habitat restriction. Like EF, but in contrast to WHG, CHG carry a variant of the *SLC24A5* gene^17^ associated with light skin colour (rs1426654, see Supplementary Note 6). This trait, which is believed to have risen to high frequency during the Neolithic expansion^18^, may thus have a relatively long history in Eurasia, with its origin probably predating the LGM.

A partial genome from a 24,000-year-old individual (MA1) from Mal'ta, Siberia^6^ had been shown to be divergent from other ancient samples and was shown by Lazaridis *et al.*^1^, using *f*_*4*_ statistics, to have more shared alleles with nearly all modern Europeans than with an EF genome. This allowed inference of an ANE component in European ancestry, which was subsequently shown to have an influence in later eastern hunter-gatherers and to have spread into Europe via an incursion of Steppe herders beginning ∼4,500 years ago^5^,^7^. Several analyses indicate that CHG genomes are not a subset of this ANE lineage. First, MA1 and CHG plot in distinct regions of the PCA and also have very different profiles in the ADMIXTURE analysis (Fig. 1). Second, when we test if CHG shows any evidence of excess allele sharing with MA1 relative to WHG using tests of the form *D*(Yoruba, *CHG*; MA1, *WHG*) no combinations were significantly positive (Supplementary Table 6). Last, we also tested whether the ancestral component inferred in modern Europeans from MA1 was distinct from any that may have been donated from CHG using tests of the form *D*(Yoruba, MA1; CHG, modern North European population) (Supplementary Table 7). All northern Europeans showed a significant sharing of alleles with MA1 separate to any they shared with CHG.

**Figure 3:**
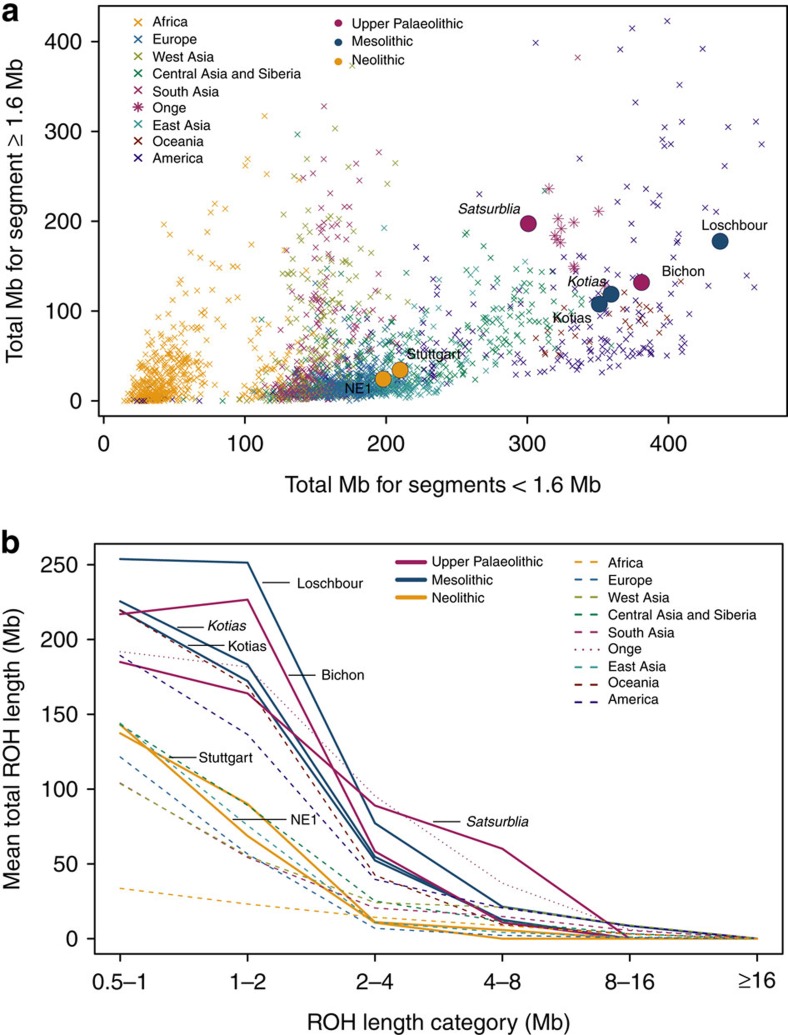
Distribution of ROH. (**a**). The total length of short ROH (<1.6 Mb) plotted against the total length of long ROH (≥1.6 Mb) and (**b**) mean total ROH length for a range of length categories. ROH were calculated using a panel of 199,868 autosomal SNPs. For Kotias we analysed both high-coverage genotypes and genotypes imputed from downsampled data (marked in italics; see Supplementary Information). Diploid genotypes imputed from low-coverage variant calls were used for Satsurblia and high-coverage genotypes were used for all other samples. A clear distinction is visible between either WHG and CHG who display an excess of shorter ROH, akin to modern Oceanic and Onge populations, and EF who resemble other populations with sustained larger ancestral population sizes.

WHG and CHG are the descendants of two ancient populations that appear to have persisted in Europe since the mid Upper Palaeolithic and survived the LGM separately. We looked at runs of homozygosity (ROH: Fig. 3) which inform on past population size^3^,^19^,^20^. Both WHG and CHG have a high frequency of ROH and in particular, the older CHG, Satsurblia, shows signs of recent consanguinity, with a high frequency of longer (>4 Mb) ROH. In contrast, EF are characterised by lower frequency of ROH of all sizes, suggesting a less constricted population history^20^,^21^, perhaps associated with a more benign passage through the LGM than the more northern populations (see Supplementary Note 7 for further details).

### Caucasus hunter-gatherer contribution to subsequent populations

**Figure 4:**
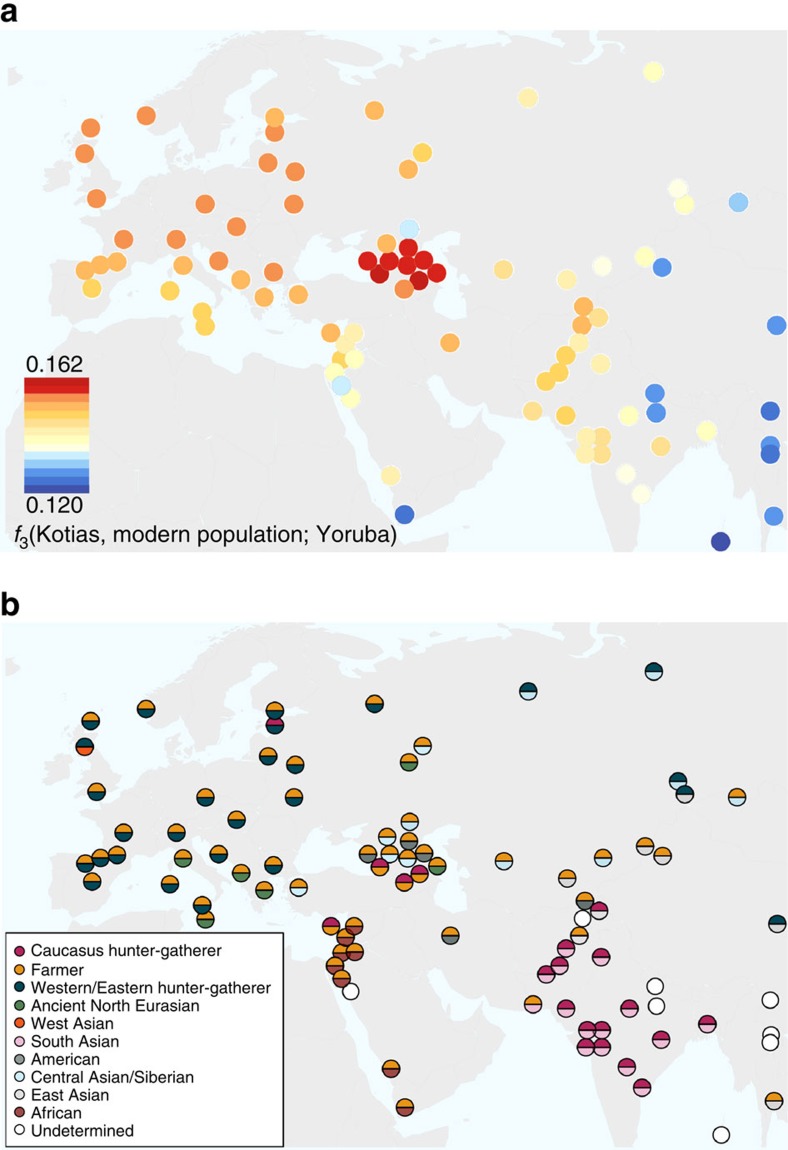
The relationship of Caucasus hunter-gatherers to modern populations. (**a**). Genomic affinity of modern populations^1^ to Kotias, quantified by the outgroup *f*_3_-statistics of the form *f*_3_(Kotias, modern population; Yoruba). Kotias shares the most genetic drift with populations from the Caucasus with high values also found for northern Europe and central Asia. (**b**). Sources of admixture into modern populations: semicircles indicate those that provide the most negative outgroup *f*_*3*_ statistic for that population. Populations for which a significantly negative statistic could not be determined are marked in white. Populations for which the ancient Caucasus genomes are best ancestral approximations include those of the Southern Caucasus and interestingly, South and Central Asia. Western Europe tends to be a mix of early farmers and western/eastern hunter-gatherers while Middle Eastern genomes are described as a mix of early farmers and Africans.

We next explored the extent to which Bichon and CHG contributed to contemporary populations using outgroup *f*_3_(African; modern, ancient) statistics, which measure the shared genetic history between an ancient genome and a modern population since they diverged from an African outgroup. Bichon, like younger WHG, shows strongest affinity to northern Europeans (Supplementary Fig. 3), while contemporary southern Caucasus populations are the closest to CHG (Fig. 4a and Supplementary Fig. 3), thus implying a degree of continuity in both regions stretching back at least 13,000 years to the late Upper Palaeolithic. Continuity in the Caucasus is also supported by the mitochondrial and Y chromosomal haplogroups of Kotias (H13c and J2a, respectively) and Satsurblia (K3 and J), which are all found at high frequencies in Georgia today^22^,^23^,^24^ (Supplementary Note 8).

EF share greater genetic affinity to populations from southern Europe than to those from northern Europe with an inverted pattern for WHG^1^,^2^,^3^,^4^,^5^. Surprisingly, we find that CHG influence is stronger in northern than Southern Europe (Fig. 4a and Supplementary Fig. 3A) despite the closer relationship between CHG and EF compared with WHG, suggesting an increase of CHG ancestry in Western Europeans subsequent to the early Neolithic period. We investigated this further using *D*-statistics of the form *D*(Yoruba, Kotias; EF, modern Western European population), which confirmed a significant introgression from CHG into modern northern European genomes after the early Neolithic period (Supplementary Fig. 4).

### CHG origins of migrating Early Bronze Age herders

**Figure 5:**
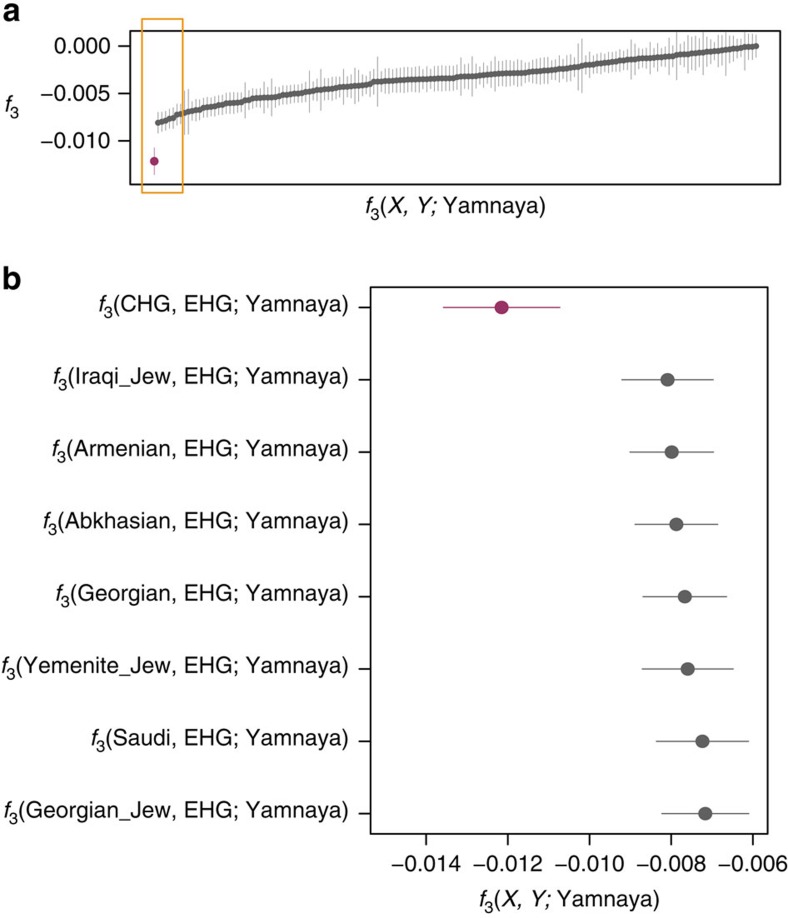
Lowest admixture *f*_3_-statistics of the form *f*_3_ (*X*, *Y*; Yamnaya). These statistics represent the Yamnaya as a mix of two populations with a more negative result signifying the more likely admixture event. (**a**). All negative statistics found for the test *f*_3_(*X*, *Y*; Yamnaya) with the most negative result *f*_3_(CHG, EHG; Yamnaya) highlighted in purple. Lines bisecting the points show the standard error. (**b**). The most significantly negative statistics which are highlighted by the yellow box in **a**. Greatest support is found for Yamnaya being a mix of Caucasus hunter-gatherers (CHG) and Russian hunter-gatherers who belong to an eastern extension of the WHG clade (EHG).

We investigated the temporal stratigraphy of CHG influence by comparing these data to previously published ancient genomes. We find that CHG, or a population close to them, contributed to the genetic makeup of individuals from the Yamnaya culture, which have been implicated as vectors for the profound influx of Pontic steppe ancestry that spread westwards into Europe and east into central Asia with metallurgy, horseriding and probably Indo-European languages in the third millenium BC^5^,^7^. CHG ancestry in these groups is supported by ADMIXTURE analysis (Fig. 1b) and admixture *f*_3_-statistics^14^,^25^ (Fig. 5), which best describe the Yamnaya as a mix of CHG and Eastern European hunter-gatherers. The Yamnaya were semi-nomadic pastoralists, mainly dependent on stock-keeping but with some evidence for agriculture, including incorporation of a plow into one burial^26^. As such it is interesting that they lack an ancestral coefficient of the EF genome (Fig. 1b), which permeates through western European Neolithic and subsequent agricultural populations. During the Early Bronze Age, the Caucasus was in communication with the steppe, particularly via the Maikop culture^27^, which emerged in the first-half of the fourth millennium BC. The Maikop culture predated and, possibly with earlier southern influences, contributed to the formation of the adjacent Yamnaya culture that emerged further to the north and may be a candidate for the transmission of CHG ancestry. In the ADMIXTURE analysis of later ancient genomes (Fig. 1b) the Caucasus component gives a marker for the extension of Yamnaya admixture, with substantial contribution to both western and eastern Bronze Age samples. However, this is not completely coincident with metallurgy; Copper Age genomes from Northern Italy and Hungary show no contribution; neither does the earlier of two Hungarian Bronze Age individuals.

### Modern impact of CHG ancestry

In modern populations, the impact of CHG also stretches beyond Europe to the east. Central and South Asian populations received genetic influx from CHG (or a population close to them), as shown by a prominent CHG component in ADMIXTURE (Supplementary Fig. 5; Supplementary Note 9) and admixture *f*_3_-statistics, which show many samples as a mix of CHG and another South Asian population (Fig. 4b; Supplementary Table 9). It has been proposed that modern Indians are a mixture of two ancestral components, an Ancestral North Indian component related to modern West Eurasians and an Ancestral South Indian component related more distantly to the Onge^25^; here Kotias proves the majority best surrogate for the former^28^,^29^ (Supplementary Table 10). It is estimated that this admixture in the ancestors of Indian populations occurred relatively recently, 1,900–4,200 years BP, and is possibly linked with migrations introducing Indo-European languages and Vedic religion to the region^28^.

## Discussion

Given their geographic origin, it seems likely that CHG and EF are the descendants of early colonists from Africa who stopped south of the Caucasus, in an area stretching south to the Levant and possibly east towards Central and South Asia. WHG, on the other hand, are likely the descendants of a wave that expanded further into Europe. The separation of these populations is one that stretches back before the Holocene, as indicated by local continuity through the Late Palaeolithic/Mesolithic boundary and deep coalescence estimates, which date to around the LGM and earlier. Several analyses show that CHG are distinct from another inferred minor ancestral population, ANE, making them a divergent fourth strand of European ancestry that expands the model of the human colonization of that continent.

The separation between CHG and both EF and WHG ended during the Early Bronze Age when a major ancestral component linked to CHG was carried west by migrating herders from the Eurasian Steppe. The foundation group for this seismic change was the Yamnaya, who we estimate to owe half of their ancestry to CHG-linked sources. These sources may be linked to the Maikop culture, which predated the Yamnaya and was located further south, closer to the Southern Caucasus. Through the Yamanya, the CHG ancestral strand contributed to most modern European populations, especially in the northern part of the continent.

Finally, we found that CHG ancestry was also carried east to become a major contributor to the Ancestral North Indian component found in the Indian subcontinent. Exactly when the eastwards movement occurred is unknown, but it likely included migration around the same time as their contribution to the western European gene pool and may be linked with the spread of Indo-European languages. However, earlier movements associated with other developments such as that of cereal farming and herding are also plausible.

The discovery of CHG as a fourth ancestral component of the European gene pool underscores the importance of a dense geographical sampling of human palaeogenomes, especially among diverse geographical regions. Its separation from other European ancestral strands ended dramatically with the extensive population, linguistic and technological upheavals of the Early Bronze Age resulting in a wide impact of this ancestral strand on contemporary populations, stretching from the Atlantic to Central and South Asia.

## Methods

### Sample preparation and DNA sequencing

DNA was extracted from three samples; two from Georgia (Kotias and Satsurblia) and one from Switzerland (Bichon; Supplementary Fig. 6). Sample preparation, DNA extraction and library construction were carried out in dedicated ancient DNA facilities at Trinity College Dublin (Kotias and Satsurblia) and the University of York, England (Bichon). DNA was extracted from Kotias and Satsurblia following a silica column based protocol^3^ based on ref. 30 and libraries were prepared and amplified with AccuPrime Pfx Supermix (Life Technology), using a modified version of ref. 31 as outlined in ref. 3. For the ancient Swiss sample Bichon, DNA was extracted following^32^ and libraries were built as described above with the exception that enzymatic end-repair was arrested using heat inactivation rather than a silica-column purification step^33^,^34^. Libraries were first screened to assess their human DNA content on an Illumina MiSeq platform at TrinSeq, Dublin using 50 base pair (bp) single-end sequencing (Supplementary Table 11). Selected libraries were further sequenced on a HiSeq 2000 platform using 100 bp single-end sequencing (Supplementary Table 12).

### Sequence processing and alignment

To reduce the effects of post-indexing contamination, raw reads were retained if the Hamming distance for the observed index was within 1 base of the expected index. Adapter sequences were trimmed from the 3' ends of reads using cutadapt version 1.3 (ref. 35), requiring an overlap of 1 bp between the adapter and the read. As ancient DNA damage is more apparent at the ends of sequences^36^,^37^, the first and last two bp of all reads from the deep sequencing phase of analysis (Supplementary Table 12) were removed using SeqTK (https://github.com/lh3/seqtk). A minimum read length of 30 bp was imposed.

Sequences were aligned using Burrows-Wheeler Aligner (BWA) version 0.7 (ref. 38), with the seed region disabled, to the GRCh37 build of the human genome with the mitochondrial sequence replaced by the revised Cambridge reference sequence (NCBI accession number NC_012920.1). Sequences from the same sample were merged using Picard MergeSamFiles (http://picard.sourceforge.net/) and duplicate reads were removed using SAMtools version 0.1.19 (ref. 39). Average depth of coverage was calculated using genome analysis toolkit (GATK) Depth of Coverage and indels were realigned using RealignerTargetCreator and IndelRealigner from the same suite of tools^40^. Reads with a mapping quality of at least 30 were retained using SAMtools^39^, and mapDamage 2.0 (ref. 41) was used with default parameters to downscale the quality scores of likely damaged bases, reducing the influence of nucleotide misincorporation on results. Only data from the deep sequencing phase of the project (100 bp single-end sequencing on a HiSeq 2,000) were used in the subsequent analyses.

### Authenticity of results

Rigorous measures were taken during laboratory work in an effort to minimize DNA contamination^3^ and negative controls were processed in parallel with samples. The authenticity of the data was further assessed *in silico* in a number of ways. The data were examined for the presence of short average sequence length and nucleotide misincorporation patterns which are characteristic of aDNA^36^,^37^ (Supplementary Figs 7 and 8). The degree of mitochondrial DNA contamination^3^,^42^ (Supplementary Table 13) and X chromosome contamination in male samples^3^ (Supplementary Tables 14-16) was also assessed (for further details see Supplementary Information).

### Molecular sex and uniparental haplogroups

Genetic sex was determined by examining the ratio of Y chromosome reads to reads aligning to both sex chromosomes^43^ (Supplementary Table 17). Mitochondrial haplogroups were assigned following^4^ with coverage determined using GATK Depth of Coverage^40^ (Supplementary Table 18). YFitter^44^, which employs a maximum likelihood based approach, was used to determine Y chromosome haplogroups for our ancient male samples (Supplementary Table 19).

### Merging ancient data with modern reference data set

Genotype calls from Kotias, Satsurblia, Bichon and selected Eurasian samples (Supplementary Table 1; Supplementary Note 10) were merged with modern genotype calls from the Human Origins data set^1^ using PLINK^45^. This data set was first filtered to exclude genotypes which had a minor allele frequency of zero in the modern populations, non-autosomal sites and modern populations with less than four individuals. Genotypes where neither allele was consistent with the GRCh37 orientation of the human genome were also removed.

For ancient samples with>8 × genome-wide coverage (namely Kotias, Bichon, Ust'-Ishim, Loschbour, Stuttgart, NE1 and BR2 (Supplementary Table 1)) genotypes were determined using GATK Unified Genotyper^40^. Genotypes were called at single-nucleotide polymorphism (SNP) positions observed in the Human Origins data set using sequencing data with a base quality≥30, depth≥8 and genotype quality≥20. The resulting VCF files were converted to PLINK format using VCFtools^46^.

For lower coverage samples genotypes were called at positions that overlapped with the Human Origins data set using GATK Pileup^40^. Bases were required to have a minimum quality of 30 and all triallelic SNPs were discarded. For SNP positions with more than one base call, one allele was randomly chosen with a probability equal to the frequency of the base at that position. This allele was duplicated to form a homozygous diploid genotype which was used to represent the individual at that SNP position^47^. This merged data set was used for PCA, ADMIXTURE, *f*_3_-statistics, *D*-statistics and ROH analysis.

### Population genetic analyses

PCA was performed by projecting selected ancient Eurasian data onto the first two principal components defined by a subset of the filtered Human Origins data set (Fig. 1a). This analysis was carried out using EIGENSOFT 5.0.1 smartpca^11^ with the lsqproject option on and the outlier removal option off. One SNP from each pair in linkage disequilibrium with *r*^2^>0.2 was removed^47^.

A clustering analysis was performed using ADMIXTURE version 1.23 (ref. 12). Genotypes were restricted to those that overlapped with the SNP capture panel described in ref. 5. Single-nucleotide polymorphisms in linkage disequilbrium were thinned using PLINK (v1.07)^45^ with parameters—indep-pairwise 200 25 0.5 (ref. 5) resulting in a set of 229,695 SNPs for analysis. Clusters (*K*) (2–20) were explored using 10 runs with fivefold cross-validation at each *K* with different random seeds (Supplementary Fig. 5). The minimal cross-validation error was found at *K*=17, but the error already starts plateauing from roughly *K*=10, implying little improvement from this point onwards. (Supplementary Fig. 9).

*D-*statistics^13^ and *f*_*3*_-statistics^14^,^25^ were used to formally assess the relationships between samples. Statistics were computed using the qpDstat (*D*-statistics) and 3PopTest (*f*_*3*_*-*statistics) programs from the ADMIXTOOLS package^14^. Significance was assessed using a block jackknife over 5 cM chunks of the genome^14^ and statistics were considered significant if their *Z*-score was of magnitude greater than 3 (ref. 25) corresponding approximately to a *P* value <0.001. For *f*_*3*_-statistics where the test population was ancient the inbreed:YES option was used.

### Dating split times using G-PhoCS

A coalescent model implemented with G-PhoCS^15^ was used to date the split among WHG, CHG and EF. Both a tree with only ancient genomes and a tree with an African San Pygmy^48^ as the outgroup were considered (see Supplementary Information for further details).

### Runs of homozygosity

Examination of ROH requires dense diploid genotypes. Imputation was used to maximise the information content of our most ancient sample, Satsurblia, following the procedure described in ref. 3. Implementing a genotype probability threshold of 0.99 (Supplementary Fig. 10), imputed data for Satsurblia and downsampled-Kotias (see Supplementary Information) was merged with high confidence diploid calls for selected ancient samples (namely Bichon, Loschbour, NE1, Stuttgart and Kotias) as well as with SNP data from modern samples using PLINK^45^. This resulted in 199,868 overlapping high-quality diploid loci for ROH analysis which was carried out using PLINK^45^ as described in ref. 3.

### Phenotypes of interest

Genes which have been associated with particular phenotypes in modern populations were examined, including some loci which have been subject to selection in European populations (Supplementary Tables 20–23). We called genotypes in Bichon, Kotias and Satsurblia using GATK Unified Genotyper^40^. For each position under investigation we only called alleles which were present in the 1,000 Genomes data set^49^, using bases with a quality≥30 in positions with a depth≥4. Because of the low average coverage of Satsurblia (1.44 × ) we also used imputed genotypes for this sample (see above) imposing a genotype probability cut-off of 0.85 (ref. 3). We used the 8-plex^50^ and Hirisplex^51^ prediction models to predict hair, eye and skin colour for our samples. Other loci investigated are discussed in the Supplementary Information.

## Additional information

**Accession codes:** Alignment data are available through the European Nucleotide Archive (ENA) under the project accession number PRJEB11364. http://www.ebi.ac.uk/ena/data/view/PRJEB11364.

**How to cite this article:** Jones, E. R. *et al.* Upper palaeolithic genomes reveal deep roots of modern eurasians. *Nat. Commun.* 6:8912 doi: 10.1038/ncomms9912 (2015).

## Supplementary Material

Supplementary Information Supplementary Figures 1-10, Supplementary Tables 1-23, Supplementary Notes 1-9 and Supplementary References

## References

[b1] Lazaridis I. *et al.* Ancient human genomes suggest three ancestral populations for present-day Europeans. Nature 513, 409–413 (2014). 25230663 10.1038/nature13673 PMC4170574

[b2] Olalde I. *et al.* Derived immune and ancestral pigmentation alleles in a 7,000-year-old Mesolithic European. Nature 507, 225–228 (2014). 24463515 10.1038/nature12960 PMC4269527

[b3] Gamba C. *et al.* Genome flux and stasis in a five millennium transect of European prehistory. Nat. Commun 5, 5257 (2014). 25334030 10.1038/ncomms6257 PMC4218962

[b4] Skoglund P. *et al.* Genomic diversity and admixture differs for stone-age Scandinavian foragers and farmers. Science 344, 747–750 (2014). 24762536 10.1126/science.1253448

[b5] Haak W. *et al.* Massive migration from the steppe was a source for Indo-European languages in Europe. Nature 522, 207–211 (2015). 25731166 10.1038/nature14317 PMC5048219

[b6] Raghavan M. *et al.* Upper Palaeolithic Siberian genome reveals dual ancestry of Native Americans. Nature 505, 87–91 (2013). 24256729 10.1038/nature12736 PMC4105016

[b7] Allentoft M. E. *et al.* Population genomics of Bronze Age Eurasia. Nature 522, 167–172 (2015). 26062507 10.1038/nature14507

[b8] Seguin-Orlando A. *et al.* Paleogenomics. Genomic structure in Europeans dating back at least 36,200 years. Science 346, 1113–1118 (2014). 25378462 10.1126/science.aaa0114

[b9] Keller A. *et al.* New insights into the Tyrolean Iceman's origin and phenotype as inferred by whole-genome sequencing. Nat. Commun. 3, 698 (2012). 22426219 10.1038/ncomms1701

[b10] Fu Q. *et al.* Genome sequence of a 45,000-year-old modern human from western Siberia. Nature 514, 445–449 (2014). 25341783 10.1038/nature13810 PMC4753769

[b11] Patterson N., Price A. L. & Reich D. Population structure and eigenanalysis. PLoS Genet. 2, e190 (2006). 17194218 10.1371/journal.pgen.0020190 PMC1713260

[b12] Alexander D. H., Novembre J. & Lange K. Fast model-based estimation of ancestry in unrelated individuals. Genome Res. 19, 1655–1664 (2009). 19648217 10.1101/gr.094052.109 PMC2752134

[b13] Green R. E. *et al.* ADraft Sequenceofthe Neandertal Genome. Science 328, 710–722 (2010). 20448178 10.1126/science.1188021 PMC5100745

[b14] Patterson N. *et al.* Ancient admixture in human history. Genetics 192, 1065–1093 (2012). 22960212 10.1534/genetics.112.145037 PMC3522152

[b15] Gronau I., Hubisz M. J., Gulko B., Danko C. G. & Siepel A. Bayesian inference of ancient human demography from individual genome sequences. Nat. Genet. 43, 1031–1034 (2011). 21926973 10.1038/ng.937 PMC3245873

[b16] Pinhasi R., Thomas M. G., Hofreiter M., Currat M. & Burger J. The genetic history of Europeans. Trends Genet. 28, 496–505 (2012). 22889475 10.1016/j.tig.2012.06.006

[b17] Lamason R. L. *et al.* SLC24A5, a putative cation exchanger, affects pigmentation in zebrafish and humans. Science 310, 1782–1786 (2005). 16357253 10.1126/science.1116238

[b18] Mathieson I. *et al.* Eight thousand years of natural selection in Europe. Preprint at http://dx.doi.org/10.1101/016477 (2015).

[b19] Morin E. Evidence for declines in human population densities during the early Upper Paleolithic in western Europe. Proc. Natl. Acad. Sci. USA 105, 48–53 (2008). 18172204 10.1073/pnas.0709372104 PMC2224228

[b20] Kirin M. *et al.* Genomic runs of homozygosity record population history and consanguinity. PLoS ONE 5, e13996 (2010). 21085596 10.1371/journal.pone.0013996 PMC2981575

[b21] Pemberton T. J. *et al.* Genomic patterns of homozygosity in worldwide human populations. Am. J. Hum. Genet. 91, 275–292 (2012). 22883143 10.1016/j.ajhg.2012.06.014 PMC3415543

[b22] Semino O. *et al.* Origin, diffusion, and differentiation of Y-chromosome haplogroups E and J: inferences on the neolithization of Europe and later migratory events in the Mediterranean area. Am. J. Hum. Genet. 74, 1023–1034 (2004). 15069642 10.1086/386295 PMC1181965

[b23] Derenko M. *et al.* Complete mitochondrial DNA diversity in Iranians. PLoS ONE 8, e80673 (2013). 24244704 10.1371/journal.pone.0080673 PMC3828245

[b24] Balanovsky O. *et al.* Parallel evolution of genes and languages in the Caucasus region. Mol. Biol. Evol. 28, 2905–2920 (2011). 21571925 10.1093/molbev/msr126 PMC3355373

[b25] Reich D., Thangaraj K., Patterson N., Price A. L. & Singh L. Reconstructing Indian population history. Nature 461, 489–494 (2009). 19779445 10.1038/nature08365 PMC2842210

[b26] Mallory J. P. *Yamna Culture*. (Encyclopedia of Indo-European Culture, 1997).

[b27] Kohl P. L. The Making of Bronze Age Eurasia Cambridge University Press (2007).

[b28] Moorjani P. *et al.* Genetic evidence for recent population mixture in India. Am. J. Hum. Genet. 93, 422–438 (2013). 23932107 10.1016/j.ajhg.2013.07.006 PMC3769933

[b29] Metspalu M. *et al.* Shared and unique components of human population structure and genome-wide signals of positive selection in South Asia. Am. J. Hum. Genet. 89, 731–744 (2011). 22152676 10.1016/j.ajhg.2011.11.010 PMC3234374

[b30] Yang D. Y., Eng B., Waye J. S., Dudar J. C. & Saunders S. R. Technical note: improved DNA extraction from ancient bones using silica-based spin columns. Am. J. Phys. Anthropol. 105, 539–543 (1998). 9584894 10.1002/(SICI)1096-8644(199804)105:4<539::AID-AJPA10>3.0.CO;2-1

[b31] Meyer M. & Kircher M. Illumina sequencing library preparation for highly multiplexed target capture and sequencing. Cold Spring Harb. Protoc. 2010, db.prot5448 (2010). 10.1101/pdb.prot5448 20516186

[b32] Rohland N., Siedel H. & Hofreiter M. A rapid column-based ancient DNA extraction method for increased sample throughput. Mol. Ecol. Resour. 10, 677–683 (2010). 21565072 10.1111/j.1755-0998.2009.02824.x

[b33] Bollongino R. *et al.* 2000 years of parallel societies in Stone Age Central Europe. Science 342, 479–481 (2013). 24114781 10.1126/science.1245049

[b34] Fortes G. G. & Paijmans J. L. A. Analysis of whole mitogenomes from ancient samples. Methods Mol. Biol. 1347, 179–195 (2015). 26374318 10.1007/978-1-4939-2990-0_13

[b35] Martin M. Cutadapt removes adapter sequences from high-throughput sequencing reads. EMBnet J. 17, 10–12 (2011).

[b36] Briggs A. W. *et al.* Patterns of damage in genomic DNA sequences from a Neandertal. Proc. Natl Acad. Sci. USA 104, 14616–14621 (2007). 17715061 10.1073/pnas.0704665104 PMC1976210

[b37] Brotherton P. *et al.* Novel high-resolution characterization of ancient DNA reveals C>U-type base modification events as the sole cause of post mortem miscoding lesions. Nucleic Acids Res. 35, 5717–5728 (2007). 17715147 10.1093/nar/gkm588 PMC2034480

[b38] Li H. & Durbin R. Fast and accurate short read alignment with Burrows-Wheeler transform. Bioinformatics 25, 1754–1760 (2009). 19451168 10.1093/bioinformatics/btp324 PMC2705234

[b39] Li H. *et al.* The Sequence Alignment/Map format and SAMtools. Bioinformatics 25, 2078–2079 (2009). 19505943 10.1093/bioinformatics/btp352 PMC2723002

[b40] McKenna A. *et al.* The Genome Analysis Toolkit: a MapReduce framework for analyzing next-generation DNA sequencing data. Genome Res. 20, 1297–1303 (2010). 20644199 10.1101/gr.107524.110 PMC2928508

[b41] Jónsson H., Ginolhac A., Schubert M., Johnson P. L. F. & Orlando L. mapDamage2.0: fast approximate Bayesian estimates of ancient DNA damage parameters. Bioinformatics 29, 1682–1684 (2013). 23613487 10.1093/bioinformatics/btt193 PMC3694634

[b42] Sánchez-Quinto F. *et al.* Genomic affinities of two 7,000-year-old Iberian hunter-gatherers. Curr. Biol. 22, 1494–1499 (2012). 22748318 10.1016/j.cub.2012.06.005

[b43] Skoglund P., Storå J., Götherström A. & Jakobsson M. Accurate sex identification of ancient human remains using DNA shotgun sequencing. J. Archaeol. Sci. 40, 4477–4482 (2013).

[b44] Jostins L. *et al.* YFitter: Maximum likelihood assignment of Y chromosome haplogroups from low-coverage sequence data. Preprint at http://arxiv.org/abs/1407.7988 (2014).

[b45] Purcell S. *et al.* PLINK: a tool set for whole-genome association and population-based linkage analyses. Am. J. Hum. Genet. 81, 559–575 (2007). 17701901 10.1086/519795 PMC1950838

[b46] Danecek P. *et al.* The variant call format and VCFtools. Bioinformatics 27, 2156–2158 (2011). 21653522 10.1093/bioinformatics/btr330 PMC3137218

[b47] Skoglund P. *et al.* Origins and genetic legacy of Neolithic farmers and hunter-gatherers in Europe. Science 336, 466–469 (2012). 22539720 10.1126/science.1216304

[b48] Prüfer K. *et al.* The complete genome sequence of a Neanderthal from the Altai Mountains. Nature 505, 43–49 (2013). 24352235 10.1038/nature12886 PMC4031459

[b49] 1000 Genomes Project Consortium. *et al.* An integrated map of genetic variation from 1,092 human genomes. Nature 491, 56–65 (2012). 23128226 10.1038/nature11632 PMC3498066

[b50] Hart K. L. *et al.* Improved eye- and skin-color prediction based on 8 SNPs. Croat. Med. J. 54, 248–256 (2013). 23771755 10.3325/cmj.2013.54.248 PMC3694299

[b51] Walsh S. *et al.* The HIrisPlex system for simultaneous prediction of hair and eye colour from DNA. Forensic Sci. Int. Genet. 7, 98–115 (2013). 22917817 10.1016/j.fsigen.2012.07.005

