# Supplementary material for: Upper Palaeolithic genomes reveal deep roots of modern Eurasians
Source: Nat Commun. 2015 Nov 16;6:8912. doi: 10.1038/ncomms9912 (PMC4660371; doi:10.1038/ncomms9912)
Supplement: Supplementary Information — Supplementary Figures 1-10, Supplementary Tables 1-23, Supplementary Notes 1-9 and Supplementary References [file ncomms9912-s1.pdf]

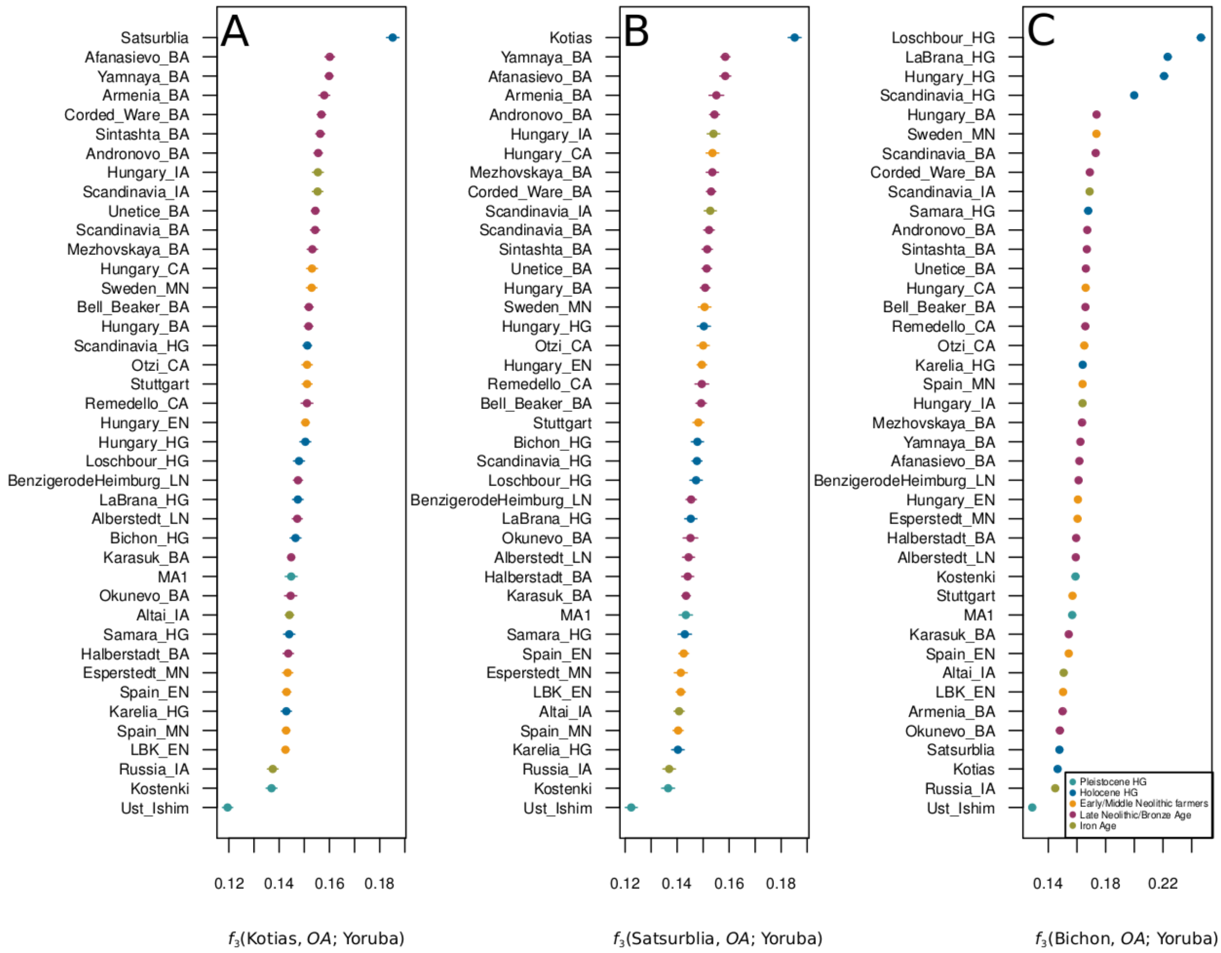

Supplementary Fig. 1. Outgroup f_3-statistics for Kotias, Satsurblia and Bichon which show the extent of shared drift with other ancient samples (OA) since they diverged from an African (Yoruban) outgroup. Satsurblia and Kotias share the most drift with each other while Bichon is closest to other western hunter-gatherers. Bars dissecting the points show standard error. HG, hunter-gatherer; EN, Early Neolithic; MN, Middle Neolithic; CA, Copper Age; BA, Bronze Age; IA, Iron Age.

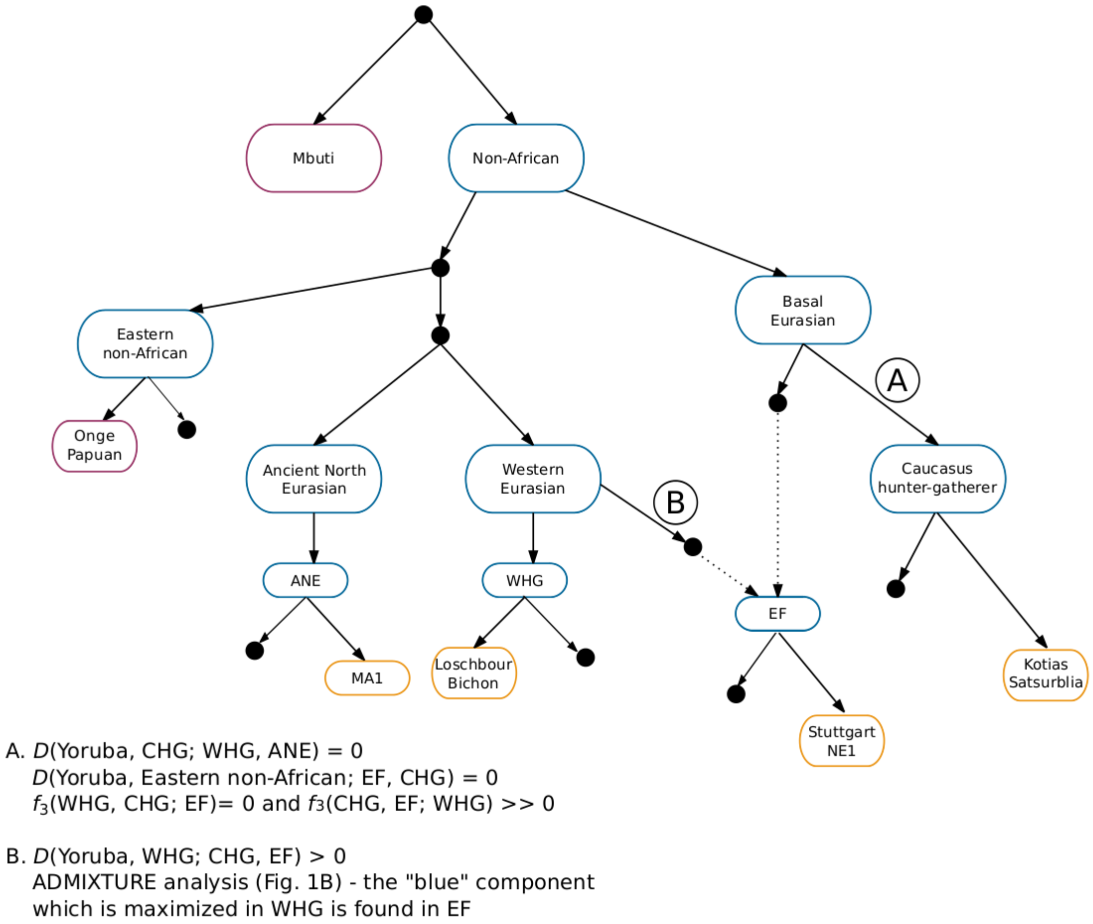

Supplementary Fig. 2. Inferred topology for ancient and modern populations. This was built on the model proposed by Lazaridis et al.[1]. **A.** Caucasus hunter-gatherers descend from a basal Eurasian branch. This is supported by both *D* and f_3 statistics (Supplementary Tables 4,6,8). **B.** Early Europeans farmers have admixed with WHG. This is supported by *D*-statistics (Supplementary Table 8) and ADMIXTURE analysis (Fig. 1B). Ancient samples are shown in yellow, inferred populations in blue and modern populations in purple. Dotted lines show descent with admixture while solid lines depict descent without admixture.

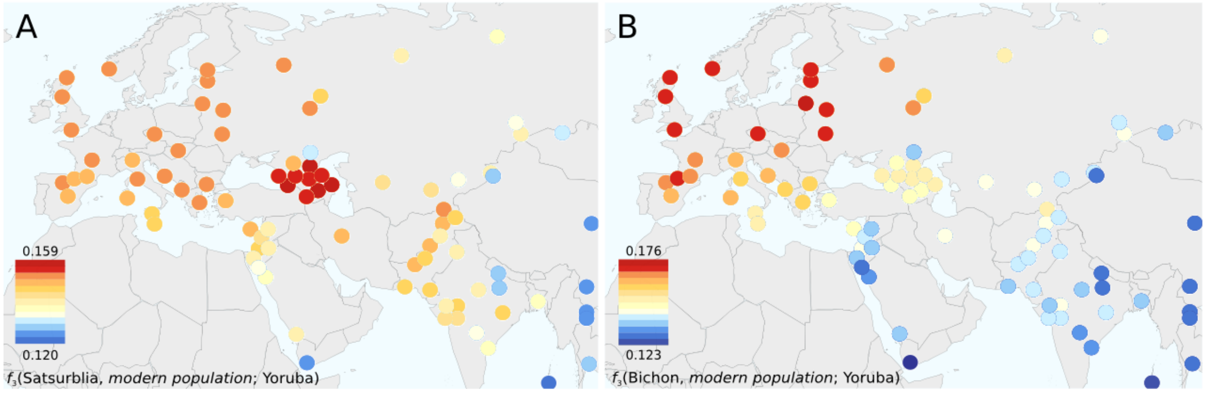

Supplementary Fig. 3. Outgroup f_3-statistics indicating the amount of shared drift between ancient samples and modern populations since they diverged from an African outgroup. Warmer colours indicate more shared drift than cooler colours. **A.** f_3(Satsurblia, *modern population*; Yoruba) which shows, similar to Kotias (Figure 4A), that Satsurblia shares the most affinity to modern populations from the Caucasus. **B.** f_3 (Bichon, *modern population*; Yoruba) which shows that Bichon shares the most drift with modern populations from Northern Europe.

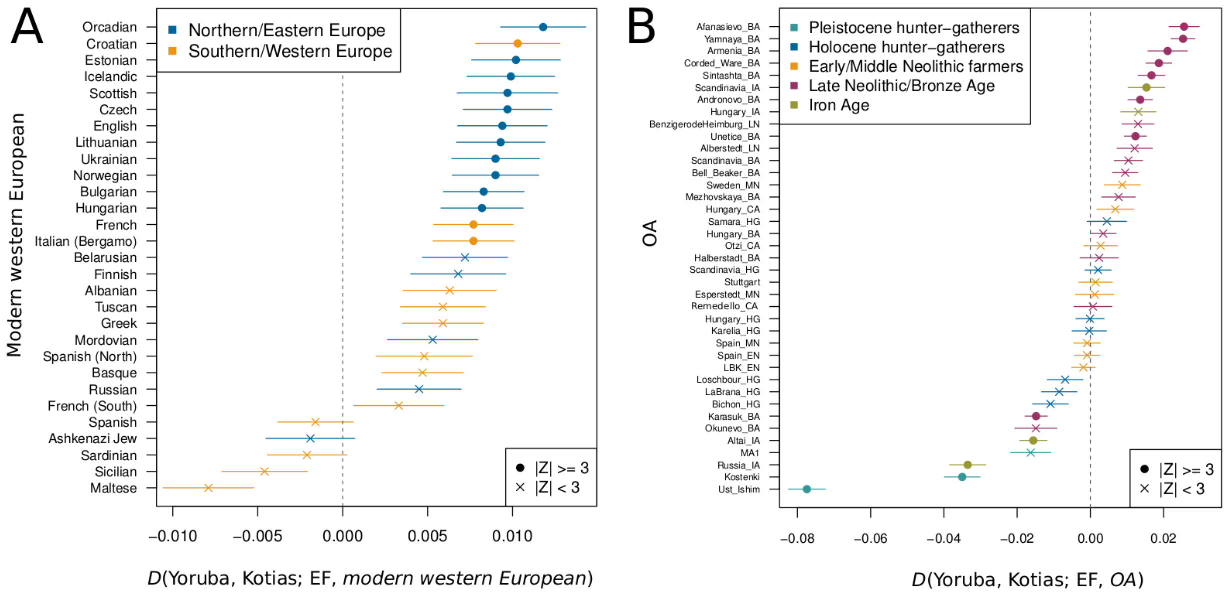

Supplementary Fig. 4. The impact of CHG on the European gene pool subsequent to the Neolithic expansion. A. *D*-statistics of the form *D*(Yoruba, Kotias; EF, *modern western European*) which suggest that there has been CHG admixture in northern/eastern Europe since the Neolithic period. Here EF are represented by Hungarian Neolithic samples[2]. **B.** *D*-statistics of the form *D*(Yoruba, Kotias; EF, *OA*) where OA represents other ancient Eurasian samples. EF are represented by Hungarian Neolithic samples[2]. Significantly positive statistics were found when OA were Late Neolithic individuals or individuals from the Yamnaya culture. These cultures may have acted as a conduit for CHG gene flow into western Europe. HG, hunter-gatherer; EN, Early Neolithic; MN, Middle Neolithic; CA, Copper Age; BA, Bronze Age; IA, Iron Age.

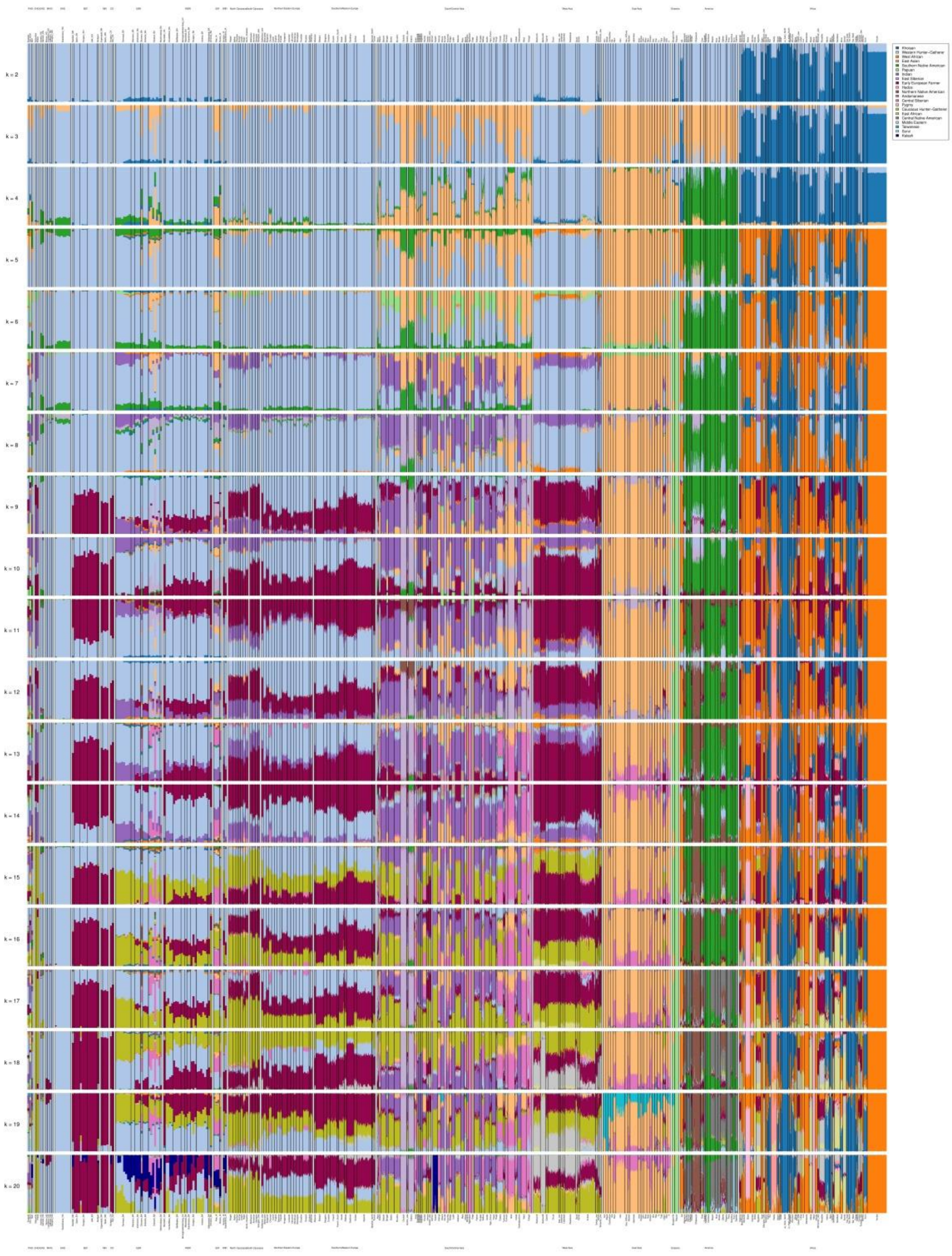

Supplementary Fig. 5. ADMIXTURE results for 2-20 clusters (*K*). Ancient samples are positioned on the left followed by modern individuals who are hierarchically clustered by population and region. Bichon resembles other western hunter-gatherers while the Caucasus hunter-gatherers Kotias and Satsurblia look unlike any other modern or ancient group.

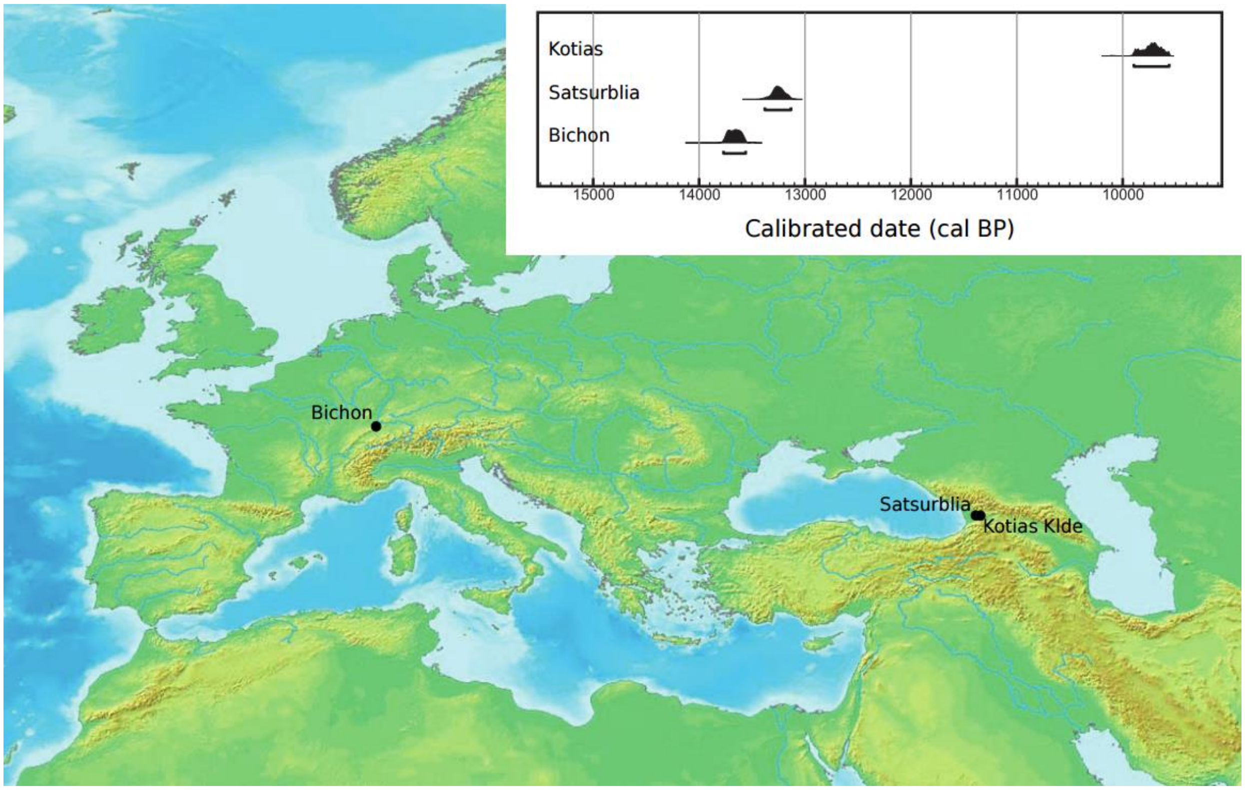

Supplementary Fig. 6. Sampling locations of Bichon (Bichon cave, Switzerland), Satsurblia (Satsurblia cave, Georgia) and Kotias (Kotias Klde cave, Georgia) accompanied by radiocarbon date curves.

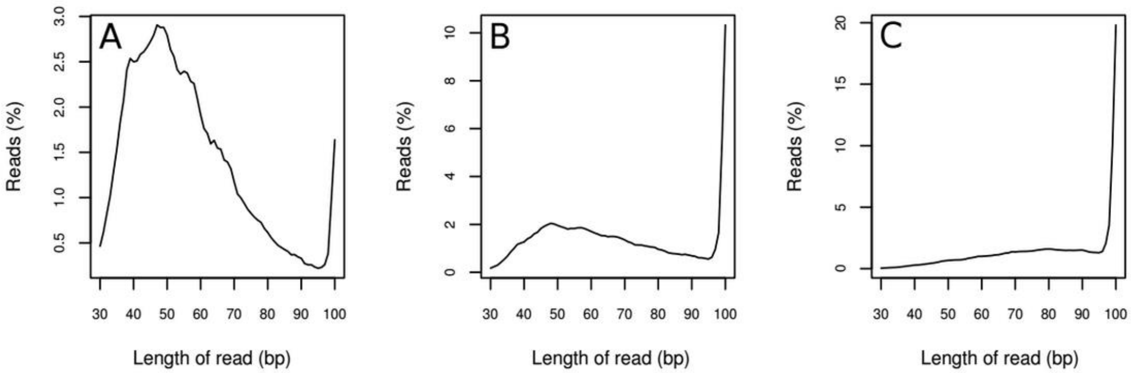

Supplementary Fig. 7. Sequence length distribution for (A) Kotias, (B) Satsurblia and (C) Bichon. All samples have sequences in the range expected for ancient DNA.

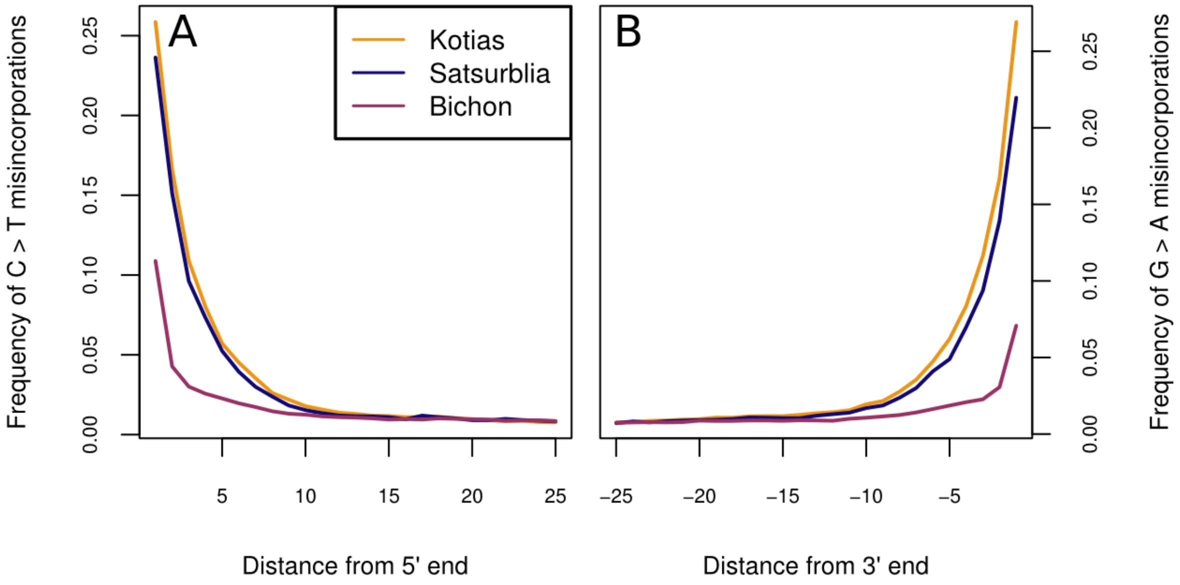

Supplementary Fig. 8. Damage patterns for Georgian and Swiss ancient samples. Plots show mismatch frequency relative to the reference genome as a function of read position. **A.** shows the frequency of C to T misincorporations at the 5' ends of reads while **B.** shows the frequency of G to A transitions at the 3' ends of reads.

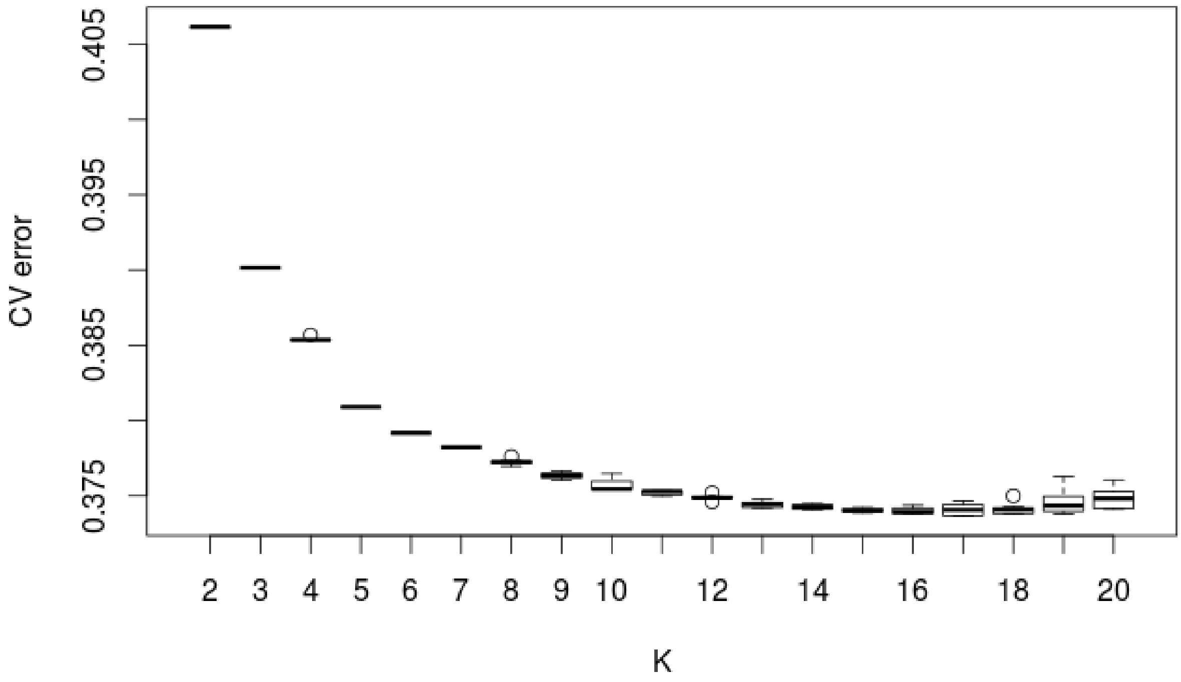

Supplementary Fig. 9. ADMIXTURE analysis cross validation (CV) error as a function of the number of clusters (*K*). The lowest mean value was attained at *K*=17.

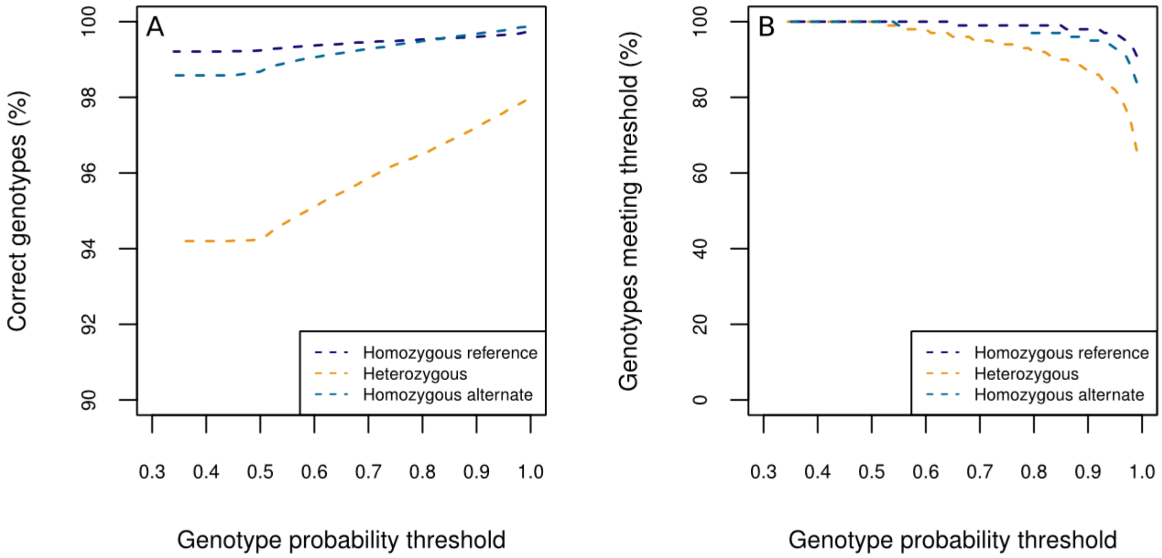

Supplementary Fig. 10. Accuracy of imputation for the Caucasus hunter-gatherer, Kotias. Genotypes are broken down into three categories - homozygous reference, heterozygous and homozygous alternate, all with respect to the reference genome. **A.** Genotypes imputed from a ~1x subsample of Kotias were compared to high coverage genotypes from the same sample and called "correct" if they matched. Heterozygous calls show the least accuracy. **B.** The proportion of genotypes retained for a range of genotype probabilities. Increasing the genotype probability threshold resulted in a reduction in the amount of genotypes meeting the threshold. These results are comparable to those found in [2].

Supplementary Table 1. Ancient data used in analyses.

Group	Reference ID	Reference	Context/Culture	Region
Hunter-gatherers				
Ust_Ishim	Ust'-Ishim	[3]	Upper Palaeolithic	Siberia
Kostenki	Kostenki	[4]	Upper Palaeolithic	Russia
MA1	Mal'ta	[5]	Upper Palaeolithic	Siberia
**Satsurblia**	**Satsurblia**	**This study**	**Upper Palaeolithic**	**Georgia**
**Bichon_HG**	**Bichon**	**This study**	**Upper Palaeolithic**	**Switzerland**
**Kotias**	**Kotias**	**This study**	**Mesolithic**	**Georgia**
Loschbour_HG	Loschbour	[1]	Mesolithic	Luxembourg
LaBrana_HG	La Braña	[6]	Mesolithic	Spain
Karelia_HG	Karelia_HG	[7]	Mesolithic	Russia
Samara_HG	Samara_HG	[7]	Neolithic hunter-gatherer	Russia
Scandinavia_HG	Motala12, I0011, I0012, I0013, I0014, I0015, I0016, Ajvide58	[1,7,8]	Mesolithic and Neolithic hunter-gatherer/ Pitted Ware Culture	Sweden
Hungary_HG	KO1	[2]	Neolithic	Hungary
Early - Middle Neolithic				
Hungary_EN	NE1, NE5, NE6, NE7	[2]	Neolithic	Hungary
Stuttgart	Stuttgart	[1]	Neolithic	Germany
LBK_EN	I0025, I0026, I0046, I0054, I0100	[7]	Linearbandkeramik	Germany
Spain_EN	I0410, I0412, I0413	[7]	Epicardial	Spain
Esperstedt_MN	I0172	[7]	Esperstedt	Germany
Spain_MN	I0406, I0407, I0408	[7]	La Mina	Spain
Sweden_MN	Gökhem2	[8]	Funnelbeaker (TRB)	Sweden
Late Neolithic – Bronze Age				
Otzi_CA	Otzi	[9]	Alpine	Italy
Hungary_CA	CO1	[2]	Baden	Hungary
Remedello_CA	RISE489	[10]	Remedello	Italy
BenzigerodeHeimburg_L[illegible]	I0058,I0059	[7]	Bell Beaker	Germany
Afanasievo_BA	RISE509, RISE511	[10]	Afanasievo	Russia
Yamnaya_BA	I0231, I0429, I0438, I0443, RISE547, RISE548, RISE550, RISE552	[7,10]	Yamnaya	Russia
Corded_Ware_BA	I0103, I0104, RISE00, RISE94	[7,10]	Corded Ware and Battle Axe	Germany/ Sweden/Estonia
Bell_Beaker_BA	I0108, I0111, I0112, RISE569	[7,10]	Bell Beaker	Germany/Czech Republic
Alberstedt_LN	I0118	[7]	Late Neolithic	Germany
Okunevo_BA	RISE516	[10]	Okunevo	Russia
Unetice_BA	I0047, I0116, I0117, I0164, RISE150, RISE577	[7,10]	Unetice	Germany/Poland/Czech Republic
Sintashta_BA	RISE392, RISE394, RISE395	[10]	Sintashta	Russia
Scandinavia_BA	RISE97, RISE98	[10]	Nordic Late Neolithic	Sweden
Andronovo_BA	RISE500, RISE503, RISE505	[10]	Andronovo	Russia
Karasuk_BA	RISE493, RISE495, RISE496, RISE497, RISE499, RISE502	[10]	Karasuk	Russia
Mezhovskaya_BA	RISE523	[10]	Mezhovskaya	Russia
Armenia_BA	RISE423	[10]	Middle Bronze Age	Armenia
Halberstadt_BA	I0099	[7]	Late Bronze Age	Germany
Hungary_BA	BR1, BR2, RISE479	[2,10]	Bronze Age	Hungary
Iron Age				
Hungary_IA	IR1	[2]	Pre-Scythian Mezőcsát	Hungary
Scandinavia_IA	RISE174	[10]	Iron Age	Sweden
Altai_IA	RISE600, RISE601, RISE602	[10]	Iron Age	Russia
Russia_IA	RISE504	[10]	Iron Age	Russia

Supplementary Table 2. *D*-statistics of the form *D*(Yoruba, *OA*; Satsurblia, Kotias) which show that Satsurblia and Kotias tend to form a clade to the exclusion of other ancient samples (|Z|<3).

OA	*D*(Yoruba,*OA*;Satsurblia,Kotias)	Z-score	P-value
Ust_Ishim	-0.0062	-1.042	0.149
Kostenki	-0.0018	-0.268	0.394
Bichon	-0.0010	-0.150	0.440
Hungary_HG	0.0016	0.233	0.408
Okunevo_BA	0.0021	0.264	0.396
Hungary_CA	0.0022	0.327	0.372
Mezhovskaya_BA	0.0028	0.456	0.324
Hungary_EN	0.0028	0.605	0.273
Halberstadt_BA	0.0032	0.450	0.326
Loschbour_HG	0.0036	0.578	0.282
Andronovo_BA	0.0040	0.845	0.199
LBK_EN	0.0042	0.889	0.187
Armenia_BA	0.0052	0.663	0.254
Hungary_BA	0.0052	1.160	0.123
MA1_HG	0.0061	0.832	0.203
Otzi_CA	0.0061	0.941	0.173
Remedello_CA	0.0066	0.880	0.189
Yamnaya_BA	0.0066	1.468	0.071
Karasuk_BA	0.0076	1.865	0.031
Samara_HG	0.0076	0.923	0.178
Russian_IA	0.0079	1.184	0.118
Spain_EN	0.0079	1.515	0.065
BenzigerodeHeimburg_LN	0.0082	1.395	0.082
Karelia_HG	0.0088	1.216	0.112
Hungary_IA	0.0089	1.278	0.101
Afanasievo_BA	0.0104	1.882	0.030
Stuttgart	0.0104	1.745	0.041
Scandinavia_BA	0.0105	1.917	0.028
Esperstedt_MN	0.0107	1.485	0.069
Spain_MN	0.0114	2.171	0.015
Bell_Beaker_BA	0.0116	2.434	0.007
Scandinavia_IA	0.0117	1.798	0.036
Sweden_MN	0.0117	1.783	0.037
Unetice_BA	0.0122	2.630	0.004
LaBrana_HG	0.0125	2.080	0.019
Alberstedt_LN	0.0133	1.877	0.030
Sweden_HG	0.0135	2.724	0.003
Corded_Ware_BA	0.0136	2.893	0.002
Altai_IA	0.0140	2.775	0.003
**Sintashta_BA**	**0.0184**	**3.627**	**1.43E-04**

OA, other ancient sample. Significant statistics are highlighted in bold.

Supplementary Table 3. *D*-statistics of the form *D*(Yoruba, *OA*; Bichon, *WHG*) which show that Bichon and western hunter-gatherers tend to form a clade to the exclusion of other ancient samples as the majority of statistics do not deviate significantly from zero (|Z|<3).

OA	D(Yoruba,OA; Bichon,Loschbour)	Z-score	P-value	D(Yoruba,OA; Bichon,La Braña)	Z-score	P-value
Afanasievo_BA	0.0073	1.059	0.145	0.0009	0.115	0.454
Alberstedt_LN	0.0142	2.607	0.005	0.0039	0.698	0.243
Altai_IA	0.0106	2.172	0.015	-0.0071	-1.467	0.071
Andronovo_BA	0.0086	1.219	0.111	0.0030	0.394	0.347
Armenia_BA	0.0133	2.710	0.003	-0.0025	-0.496	0.310
Bell_Beaker_BA	0.0073	1.777	0.038	-0.0003	-0.069	0.472
BenzigerodeHeimburg_LN	0.0071	1.094	0.137	-0.0007	-0.103	0.459
Corded_Ware_BA	0.0137	1.806	0.036	0.0197	2.384	0.009
Esperstedt_MN	0.0121	1.669	0.048	-0.0036	-0.469	0.320
Halberstadt_BA	0.0081	1.418	0.078	-0.0012	-0.208	0.418
Hungary_BA	0.0082	1.516	0.065	-0.0055	-1.005	0.157
Hungary_CA	0.0135	2.701	0.003	-0.0014	-0.259	0.398
Hungary_EN	0.0136	2.251	0.012	0.0035	0.544	0.293
Hungary_HG	0.0126	1.831	0.034	0.0018	0.240	0.405
Hungary_IA	0.0048	1.041	0.149	-0.0033	-0.667	0.252
Karasuk_BA	0.0067	0.923	0.178	-0.0096	-1.187	0.118
Karelia_HG	0.0132	1.819	0.035	-0.0081	-1.050	0.147
Kostenki	0.0105	2.301	0.011	0.0052	1.071	0.142
Kotias	0.0101	1.187	0.118	-0.0346	-4.079	0.000
LBK_EN	0.0033	0.481	0.315	-0.0095	-1.289	0.099
MA1_HG	0.0054	1.140	0.127	-0.0001	-0.019	0.492
Mezhovskaya_BA	0.0098	1.537	0.062	0.0015	0.215	0.415
Okunevo_BA	0.0139	2.132	0.017	-0.0061	-0.896	0.185
Otzi_CA	0.0163	2.244	0.012	0.0104	1.335	0.091
Remdello_CA	0.0075	1.200	0.115	0.0044	0.690	0.245
Russia_IA	0.0045	0.660	0.255	-0.0040	-0.579	0.281
Samara_HG	0.0101	2.055	0.020	0.0046	0.937	0.174
Satsurblia	0.0053	0.714	0.238	0.0015	0.207	0.418
Scandinavia_BA	0.0036	0.544	0.293	-0.0003	-0.049	0.480
Scandinavia_IA	0.0031	0.376	0.353	-0.0039	-0.467	0.320
Sintashta_BA	0.0036	0.531	0.298	-0.0069	-0.993	0.160
Spain_EN	0.0063	1.203	0.114	0.0024	0.423	0.336
Spain_MN	0.0104	1.926	0.027	0.0028	0.500	0.309
Stuttgart	0.0131	2.212	0.014	-0.0041	-0.693	0.244
Sweden_HG	**0.0236**	**4.463**	**4.04E-06**	**-0.0185**	**-3.439**	**2.92E-04**
Sweden_MN	0.0111	1.669	0.048	-0.0025	-0.350	0.363
Unetice_BA	**0.0154**	**3.371**	**3.74E-04**	-0.0008	-0.159	0.437
Ust_Ishim	0.0065	1.523	0.064	-0.0021	-0.451	0.326
Yamnaya_BA	0.0055	0.891	0.186	0.0013	0.205	0.419

OA, other ancient sample. Significant statistics are highlighted in bold.

Supplementary Table 4. f_3-statistics which elucidate the topology between CHG, WHG and EF.

Tree	Pop1	Pop2	Outgroup	f_3	Standard error	Z-score
f_3(CHG, EF; WHG) 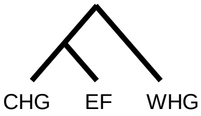	Kotias	NE1	Bichon	0.105	0.007	14.493
	Kotias	Stuttgart	Bichon	0.111	0.007	15.616
	Kotias	NE1	Loschbour	0.151	0.008	18.273
	Kotias	Stuttgart	Loschbour	0.161	0.008	19.018
f_3(WHG, EF; CHG) 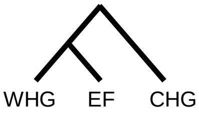	Bichon	NE1	Kotias	0.055	0.006	9.480
	Loschbour	NE1	Kotias	0.061	0.006	10.206
	Bichon	Stuttgart	Kotias	0.049	0.006	8.543
	Loschbour	Stuttgart	Kotias	0.052	0.006	9.285
f_3(WHG, CHG; EF) 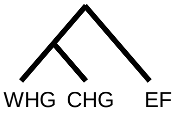	Kotias	Bichon	NE1	0.010	0.004	2.384
	Kotias	Loschbour	NE1	0.006	0.004	1.489
	Kotias	Bichon	Stuttgart	0.018	0.004	4.019
	Kotias	Loschbour	Stuttgart	0.015	0.004	3.313

Supplementary Table 5. Parameters estimates from G-PhoCS.

Parameter	Stuttgart, San	Stuttgart, no outgroup	NE1, San	NE1, no outgroup
θ_{San}	69,578 (28,507–149,950)		65,565 (28,937–140,883)	
$\theta_{Loschbour}$	8,514 (1,242–27,458)	8,714 (1,230–27,819)	8201 (1058–27105)	8,294 (1,179–27,274)
$\theta\tau_{Bichon}$	7,641 (939–26,320)	7,676 (799–26,376)	7,896 (817–26,525)	7,648 (742–26,287)
$\theta\tau_{Kotias}$	7,267 (1,096–24,384)	7,208 (1,182–25,058)	5,098 (1,206–17,066)	4,672 (899–18,650)
θ_{EF}	8,996 (1,810–27,407)	7,715 (1,207–25,545)	9,273 (2,199–27,020)	8,037 (1,706–25,676)
$\theta_{Loschbour+Bichon}$	4,240 (1,201–10,339)	1,824 (390–4,593)	4,686 (1,562–10,541)	2,296 (611–5,428)
$\theta_{Kotias+EF}$	5,313 (913–16,765)	2613 (607–11,510)	8,570 (1,227–25,662)	7,176 (966–23,764)
$\theta_{Loschbour+Bichon+Kotias+EF}$	8312 (4,622–11,876)	13,086 (11,690–14,642)	9,214 (5,642–12,959)	13,021 (11,614–14,524)
$\theta_{Loschbour+Bichon+Kotias+EF+San}$	15,567 (13,408–17,961)		15,103 (13,007–17,381)	
$\tau_{Loschbour+Bichon}$	17,616 (13,993–28,855)	18,115 (13,955–29,124)	17,235 (13,917–27,110)	16,729 (13,893–24,470)
$\tau_{Kotias+EF}$	23,962 (11,439–43,045)	20,360 (12,607–32,806)	31,800 (17,814–53,626)	22,990 (11,510–39,768)
$\tau_{Loschbour+Bichon+Kotias+EF}$	46,441 (27,010–75,773)	33,806 (21,634–52,601)	52171 (30,555–84,651)	37,341 (21,923–61,832)
$\tau_{Loschbour+Bichon+Kotias+EF+San}$	233,172 (157,714–309,530)		259,090 (189,055–335,442)	
$m_{Loschbour\text{->}EF}$	230,977 (20,299–778,204)	282,893 (20,052–877,763)	200,098 (19,826–700,477)	210,978 (16,845–728,345)
$propm_{Loschbour\text{->}EF}$	0.09 (0.01–0.34)	0.12 (0.01–0.39)	0.08 (0.01–0.31)	0.07 (0.01–0.28)

Mean and 95% HDP intervals for models with four possible trees, with EF represented by either Stuttgart or NE1, and with or without an outgroup (San).

Supplementary Table 6. *D*-statistics of the form *D*(Yoruba, *CHG*; *WHG*, *OA*) which show that western hunter-gatherers and eastern hunter-gatherers as well as the latter and the ancient north Eurasian MA1 form a clade to the exclusion of CHG (|Z|<3).

WHG	OA	*D*(Yoruba, Kotias; *WHG*, *OA*)	Z-score	P-value	D(Yoruba, Satsurblia; *WHG*, *OA*)	Z-score	P-value
Bichon	MA1	-0.0067	-1.002	0.158	-0.0114	-1.498	0.067
Hungary_HG	MA1	-0.0203	-2.831	0.002	-0.0218	-2.595	0.005
La Braña	MA1	-0.0092	-1.364	0.086	-0.0078	-1.085	0.139
Loschbour	MA1	-0.0102	-1.606	0.054	-0.0135	-1.888	0.030
Bichon	Samara_HG	0.0080	1.195	0.116	0.0129	1.554	0.060
Hungary_HG	Samara_HG	0.0039	0.517	0.303	0.0011	0.111	0.456
La Braña	Samara_HG	0.0131	1.913	0.028	0.0100	1.222	0.111
Loschbour	Samara_HG	0.0130	1.864	0.031	0.0119	1.515	0.065
Bichon	Karelia_HG	0.0136	2.188	0.014	0.0035	0.446	0.328
Hungary_HG	Karelia_HG	-0.0019	-0.270	0.394	-0.0079	-0.941	0.173
La Braña	Karelia_HG	0.0087	1.379	0.084	0.0035	0.479	0.316
Loschbour	Karelia_HG	0.0089	1.447	0.074	0.0008	0.106	0.458

WHG, western hunter-gatherer; OA, other ancient sample.

Supplementary Table 7. CHG and ANE ancestry in distinct is modern Northern Europeans.

Modern Northern European	*D*(Yoruba, MA1; Kotias, *modern Northern European*)	Z-score	P-value
**Belarusian**	**0.0286**	**5.693**	**6.24E-09**
**English**	**0.0253**	**4.965**	**3.44E-07**
**Estonian**	**0.0351**	**6.941**	**1.95E-12**
**Finnish**	**0.0331**	**6.392**	**8.19E-11**
**Icelandic**	**0.0278**	**5.516**	**1.73E-08**
**Lithuanian**	**0.034**	**6.534**	**3.20E-11**
**Mordovian**	**0.0314**	**6.297**	**1.52E-10**
**Norwegian**	**0.0271**	**5.423**	**2.93E-08**
**Orcadian**	**0.0267**	**5.365**	**4.05E-08**
**Russian**	**0.0307**	**6.221**	**2.47E-10**
**Scottish**	**0.0285**	**5.532**	**1.58E-08**

Significant statistics are highlighted in bold.

Supplementary Table 8. D-statistics of the form D(Yoruba, *X*; CHG, EF) which show that CHG and EF do not form a clade to the exclusion of WHG (Z >3) but do form a clade to the exclusion of MA1 and eastern non-Africans (|Z| < 3).

X	CHG	EF	*D*(Yoruba,*X*;*CHG*,*EF*)	Z-score	P-value
**Bichon**	**Kotias**	**Stuttgart**	**0.0244**	**3.902**	**4.77E-05**
**Bichon**	**Kotias**	**NE1**	**0.0336**	**5.450**	**2.52E-08**
**Bichon**	**Satsurblia**	**Stuttgart**	**0.0229**	**3.622**	**1.46E-04**
**Bichon**	**Satsurblia**	**NE1**	**0.0351**	**5.433**	**2.77E-08**
**La Braña**	**Kotias**	**Stuttgart**	**0.0216**	**3.711**	**1.03E-04**
**La Braña**	**Kotias**	**NE1**	**0.0362**	**6.203**	**2.77E-10**
**La Braña**	**Satsurblia**	**Stuttgart**	**0.0277**	**4.262**	**1.01E-05**
**La Braña**	**Satsurblia**	**NE1**	**0.0447**	**7.050**	**8.95E-13**
**Loschbour**	**Kotias**	**Stuttgart**	**0.0341**	**5.737**	**4.82E-09**
**Loschbour**	**Kotias**	**NE1**	**0.0477**	**7.815**	**2.75E-15**
**Loschbour**	**Satsurblia**	**Stuttgart**	**0.0369**	**5.834**	**2.71E-09**
**Loschbour**	**Satsurblia**	**NE1**	**0.0520**	**8.185**	**1.36E-16**
MA1	Kotias	Stuttgart	-0.0153	-2.380	0.009
MA1	Kotias	NE1	-0.0083	-1.362	0.087
MA1	Satsurblia	Stuttgart	-0.0096	-1.423	0.077
MA1	Satsurblia	NE1	-0.0006	-0.084	0.467
Onge	Kotias	NE1	-0.0024	-0.558	0.288
Onge	Kotias	Stuttgart	-0.0030	-0.678	0.249
Onge	Satsurblia	NE1	0.0009	0.202	0.420
Onge	Satsurblia	Stuttgart	-0.0004	-0.085	0.466
Papuan	Kotias	NE1	-0.0009	-0.199	0.421
Papuan	Kotias	Stuttgart	0.0021	0.454	0.325
Papuan	Satsurblia	NE1	0.0001	0.029	0.488
Papuan	Satsurblia	Stuttgart	0.0019	0.393	0.347

Significant statistics are highlighted in bold.

Supplementary Table 9. Lowest statistics for the test f_3(*Target; Source$_1$, Source$_2$*).

Target	Region of target	Source$_1$	Source$_2$	Lowest f_3	Standard error	Z-score
Altaian	Central Asia/Siberia	**Scandinavia_HG (I0013)**	Ulchi	-0.020	0.001	-23.378
Kalmyk	Central Asia/Siberia	**LBK_EN (I0054)**	Ulchi	-0.017	0.001	-22.109
Kyrgyz	Central Asia/Siberia	**Spain_EN (I0410)**	Ulchi	-0.025	0.001	-30.837
Mansi	Central Asia/Siberia	**Scandinavia_HG (I0015)**	Nganasan	-0.021	0.001	-17.357
Selkup	Central Asia/Siberia	**Scandinavia_HG (I0015)**	Nganasan	-0.023	0.001	-20.470
Tajik Pomiri	Central Asia/Siberia	**LBK_EN (I0026)**	Karitiana	-0.012	0.001	-8.257
Tubalar	Central Asia/Siberia	**Scandinavia_HG (Motala12)**	Korean	-0.012	0.001	-12.045
Turkmen	Central Asia/Siberia	**LBK_EN (I0046)**	Nganasan	-0.025	0.001	-20.594
Tuvinian	Central Asia/Siberia	**LBK_EN (I0046)**	Nganasan	-0.012	0.001	-12.131
Uzbek	Central Asia/Siberia	**LBK_EN (I0046)**	Hezhen	-0.027	0.001	-27.451
Dai	*East Asia*	***Hungary_EN (NE6)***	*Ami*	*0.000*	*0.001*	*-0.074*
Lahu	*East Asia*	***LBK_EN (I0046)***	*Dai*	*0.014*	*0.001*	*13.381*
Naxi	*East Asia*	***Spain_EN (I0410)***	*She*	*0.001*	*0.001*	*1.429*
Thai	East Asia	**LBK_EN (I0026)**	Ami	-0.005	0.001	-4.801
Tu	East Asia	**Scandinavia_HG (Motala12)**	Korean	-0.009	0.001	-10.881
Uygur	East Asia	**LBK_EN (I0046)**	She	-0.028	0.001	-30.514
Xibo	East Asia	**LBK_EN (I0025)**	Korean	-0.007	0.001	-6.628
Bulgarian	Eastern Europe	**Hungary_EN (NE6)**	**Samara_HG**	-0.015	0.002	-6.927
Czech	Eastern Europe	**Hungary_EN (NE6)**	**Samara_HG**	-0.016	0.002	-6.924
Hungarian	Eastern Europe	**Hungary_EN (NE6)**	**Samara_HG**	-0.015	0.002	-6.804
Ukrainian	Eastern Europe	**Hungary_EN (NE6)**	**Samara_HG**	-0.015	0.002	-6.339
Adygei	North Caucasus	**LBK_EN (I0026)**	Karitiana	-0.008	0.002	-5.094
Balkar	North Caucasus	**LBK_EN (I0046)**	Nganasan	-0.012	0.001	-9.455
Chechen	North Caucasus	**LBK_EN (I0100)**	Karitiana	-0.007	0.001	-4.741
Kumyk	North Caucasus	**LBK_EN (I0100)**	Karitiana	-0.013	0.001	-8.589
Lezgin	North Caucasus	**LBK_EN (I0046)**	**MA1**	-0.010	0.002	-4.737
Nogai	North Caucasus	**LBK_EN (I0046)**	Nganasan	-0.024	0.001	-21.062
North Ossetian	North Caucasus	**LBK_EN (I0046)**	Nganasan	-0.011	0.001	-8.067
Belarusian	Northern Europe	**Hungary_EN (NE6)**	**Samara_HG**	-0.014	0.002	-6.121
Chuvash	Northern Europe	**LBK_EN (I0046)**	Nganasan	-0.021	0.001	-17.147
English	Northern Europe	**Hungary_EN (NE6)**	**Samara_HG**	-0.015	0.002	-6.330
Estonian	Northern Europe	**Kotias**	**Loschbour**	-0.012	0.002	-7.432
Finnish	Northern Europe	**Hungary_EN (NE6)**	**Samara_HG**	-0.012	0.002	-4.709
Icelandic	Northern Europe	**Hungary_EN (NE6)**	**Samara_HG**	-0.013	0.002	-5.491
Lithuanian	Northern Europe	**Hungary_EN (NE6)**	**Samara_HG**	-0.012	0.002	-5.106
Mordovian	Northern Europe	**LBK_EN (I0100)**	**MA1**	-0.013	0.002	-5.958
Norwegian	Northern Europe	**Hungary_EN (NE6)**	**Samara_HG**	-0.013	0.002	-5.562
Orcadian	Northern Europe	**Hungary_EN (NE6)**	**Samara_HG**	-0.013	0.002	-5.527
Russian	Northern Europe	**Hungary_EN (NE6)**	**Samara_HG**	-0.013	0.002	-6.125
Scottish	Northern Europe	**Loschbour**	Iraqi Jew	-0.011	0.001	-8.567
Balochi	South Asia	**Kotias**	Kharia	-0.006	0.001	-7.351
Bengali	South Asia	**Kotias**	Kharia	-0.011	0.001	-13.990
Brahui	South Asia	**Kotias**	Kharia	-0.004	0.001	-5.154
Burusho	South Asia	**Satsurblia**	Korean	-0.012	0.001	-11.349
GujaratiA	South Asia	**Kotias**	Kharia	-0.011	0.001	-12.005
GujaratiB	South Asia	**Kotias**	Kharia	-0.012	0.001	-12.427
GujaratiC	South Asia	**Kotias**	Kharia	-0.010	0.001	-10.205
GujaratiD	South Asia	**Kotias**	Kharia	-0.007	0.001	-7.217
Hazara	South Asia	**Spain_EN (I0410)**	Korean	-0.025	0.001	-27.520
Iranian	South Asia	**LBK_EN (I0026)**	Guarani	-0.011	0.001	-7.943
Kalash	*South Asia*	***Kotias***	*Cabecar*	*0.016*	*0.001*	*11.166*
Kharia	*South Asia*	*Lahu*	*Mala*	*0.001*	*0.000*	*2.675*
Kusunda	*South Asia*	***LBK_EN (I0054)***	*Naxi*	*0.010*	*0.001*	*9.149*
Lodhi	South Asia	**Kotias**	Kharia	-0.008	0.001	-10.559
Makrani	South Asia	**LBK_EN (I0046)**	Vishwabrahmin	-0.004	0.001	-5.672
Mala	South Asia	**Kotias**	Kharia	-0.007	0.001	-10.147
Onge	*South Asia*	*Cambodian*	*Papuan*	*0.130*	*0.002*	*61.297*
Pathan	South Asia	**Kotias**	Kharia	-0.011	0.001	-13.879
Punjabi	South Asia	**Kotias**	Kharia	-0.010	0.001	-12.115
Sindhi	South Asia	**Kotias**	Kharia	-0.012	0.001	-15.623
Tiwari	South Asia	**Kotias**	Kharia	-0.012	0.001	-17.322
Vishwabrahmin	South Asia	**Kotias**	Kharia	-0.008	0.001	-12.198
Abkhasian	South Caucasus	**Kotias**	**LBK_EN (I0046)**	-0.010	0.002	-5.847
Armenian	South Caucasus	**Satsurblia**	**LBK_EN (I0046)**	-0.010	0.002	-5.040
Georgian	South Caucasus	**Satsurblia**	**LBK_EN (I0046)**	-0.011	0.002	-5.469
Albanian	Southern Europe	**LBK_EN (I0046)**	**MA1**	-0.013	0.002	-5.833
Basque	Southern Europe	**LBK_EN (I0026)**	**Bichon**	-0.009	0.002	-4.861
Croatian	Southern Europe	**Hungary _EN (NE6)**	**Samara_HG**	-0.015	0.002	-6.644
Greek	Southern Europe	**LBK_EN (I0100)**	**MA1**	-0.013	0.002	-6.343
Italian (Bergamo)	Southern Europe	**Hungary _EN (NE6)**	**Samara_HG**	-0.013	0.002	-5.762
Maltese	Southern Europe	**LBK_EN (I0100)**	**MA1**	-0.010	0.002	-4.834
Sardinian	Southern Europe	**LBK_EN (I0026)**	**Bichon**	-0.007	0.002	-3.666
Sicilian	Southern Europe	**Hungary _EN (NE6)**	**Samara_HG**	-0.012	0.002	-5.216
Spanish	Southern Europe	**Hungary _EN (NE6)**	**Samara_HG**	-0.014	0.002	-6.580
Spanish (North)	Southern Europe	**Hungary _EN (NE6)**	**Scandinavia_HG (I0012)**	-0.013	0.002	-5.526
Tuscan	Southern Europe	**LBK_EN (I0100)**	**MA1**	-0.011	0.002	-5.366
BedouinA	West Asia	**LBK_EN (I0046)**	Mende	-0.019	0.001	-18.211
BedouinB	*West Asia*	***LBK_EN (I0046)***	*Mende*	*0.005*	*0.001*	*4.030*
Cypriot	West Asia	**Satsurblia**	**LBK_EN (I0046)**	-0.008	0.002	-3.774
Druze	West Asia	**LBK_EN (I0046)**	Dinka	-0.005	0.001	-4.151
Jordanian	West Asia	**LBK_EN (I0046)**	Dinka	-0.016	0.001	-13.288
Lebanese	West Asia	**Spain_EN (I0410)**	Luo	-0.013	0.001	-12.029
Palestinian	West Asia	**LBK_EN (I0046)**	Dinka	-0.014	0.001	-13.743
Saudi	West Asia	**LBK_EN (I0046)**	Dinka	-0.007	0.001	-5.134
Syrian	West Asia	**LBK_EN (I0046)**	Mende	-0.013	0.001	-10.934
Turkish	West Asia	**LBK_EN (I0046)**	Nganasan	-0.015	0.001	-13.709
Yemen	West Asia	**Spain_EN (I0410)**	Mende	-0.025	0.001	-21.821
French	Western Europe	**Hungary _EN (NE6)**	**Samara_HG**	-0.015	0.002	-6.810
French (South)	Western Europe	**Hungary _EN (NE6)**	**Scandinavia_HG (I0012)**	-0.012	0.002	-5.624

Ancient Samples are highlighted in bold. Populations for which source populations could not be determined (Z > -3) are italicized.

Supplementary Table 10. Highest *D*-statistics for *D*(Yoruba, *X*; Onge, *Indian population*) where we let X be every possible non-African population and ancient sample in the Human Origins dataset.

Indian population	X	*D*(Yoruba, *X*; Onge, *Indian population*)	Z-score	P-value
GujaratiC	Kotias	0.0540	15.925	2.13E-57
GujaratiD	Kotias	0.0503	15.311	3.23E-53
Lodhi	Kotias	0.0448	14.829	4.76E-50
Mala	Kotias	0.0368	12.339	2.79E-35
Vishwabrahmin	Kotias	0.0393	13.025	4.41E-39
GujaratiA	Afanasievo_BA	0.0623	20.594	1.55E-94
GujaratiB	Afanasievo_BA	0.0576	20.032	1.45E-89
Tiwari	Afanasievo_BA	0.0602	23.159	5.90E-119
Kharia	Mala	0.0240	13.062	2.71E-39

Supplementary Table 11.Summary of the results for all samples sequenced during the screening phase of the project.

Sample ID	Lab ID	Skeletal element	Mass of powder (g)	Total reads	Aligned non-clonal reads	Human DNA (%)
**Bichon**	**Bichon**	**Petrous**	**0.250**	**3,501,019**	**2,504,728**	**71.54**
Kotias	KK1.C1	Molar Crown	0.335	584,353	385,012	65.89
**Kotias**	**KK1.R1**	**Molar Root**	**0.335**	**255,035**	**196,060**	**76.88**
Satsurblia	SATP.1	Petrous	0.373	3,333,552	307,444	9.22
**Satsurblia**	**SATP.3**	**Petrous**	**0.372**	**2,228,960**	**306,748**	**13.76**

Samples were sequenced using 50 base pair (bp) single end sequencing on a MiSeq platform. Adapter trimmed reads were aligned to the GRCh37 build of the human genome with the mitochondrial sequence replaced by the revised Cambridge reference sequence and clonal reads were removed using SAMtools (see methods). Samples highlighted in bold were selected for further sequencing.

Supplementary Table 12. Summary of the results for samples sequenced during the deep sequencing phase of the project.

Sample ID	Lab ID	Sequencing facility	Number of lanes	Total reads	Aligned non-clonal reads	Human DNA (%)	Coverage (x)
Bichon	Bichon	BGI*	7	1,139,300,766	352,921,957	30.98	9.50
Kotias	KK1.R1	BC^	6	1,327,399,258	849,496,700	64.00	15.38
Satsurblia	SATP.3	BC^	3	419,683,577	66,338,333	15.81	1.44

* Beijing Genomics Institute, China; ^ Beckmann Coulter Inc., USA

All sequencing was performed on a HiSeq 2000 platform using 100 bp single end sequencing. Adapter trimmed reads were aligned to the GRCh37 build of the human genome with the mitochondrial sequence replaced by the revised Cambridge reference sequence and clonal reads were removed using SAMtools. Coverage was calculated using GATK (see methods).

Supplementary Table 13. Mitochondrial contamination estimates.

Sample	%C	%C+MD
Kotias	0.07	0.32
Satsurblia	0.11	0.57
Bichon	0.16	0.62

%C, percentage contamination excluding sites with potentially damaged bases; %C + MD, percentage contamination including sites with potentially damaged bases

Estimates are derived from the proportion of secondary bases at haplogroup-defining positions in the mitochondrial genome.

Supplementary Table 14. Establishing the background error rate for X chromosome contamination estimates.

Test	Sample	Allele	-4	-3	-2	-1	0	1	2	3	4
1	Kotias	p	1,856,365	1,854,061	1,849,192	1,842,313	1,773,694	1,841,046	1,847,342	1,853,194	1,855,144
	Kotias	s	4,582	4,483	4,593	5,453	10,001	5,570	4,574	4,497	4,525
	Satsurblia	p	36,003	36,133	36,182	35,863	34,645	35,978	36,204	36,022	36,293
	Satsurblia	s	90	57	84	97	155	86	61	72	68
	Bichon	p	980,753	980,819	979,916	978,924	948,648	978,001	979,943	980,432	980,784
	Bichon	s	2,458	2,411	2,429	2,743	4,164	2,692	2,391	2,457	2,504
2	Kotias	p	223,214	223,081	222,624	222,014	214,334	221,873	222,412	222,897	223,050
	Kotias	s	618	519	567	651	1215	657	559	531	559
	Satsurblia	p	11,201	11,238	11,264	11,171	10,793	11,201	11,269	11,202	11,291
	Satsurblia	s	30	22	23	33	44	25	14	28	17
	Bichon	p	143,919	143,956	143,754	143,676	139,533	143,524	143,777	143,841	143,950
	Bichon	s	319	343	370	422	616	387	360	365	355

“Test 1” and “test 2” were performed as in [11]. The number of primary alleles (p) and secondary alleles (s) in ancient male samples at X chromosome sites found to be polymorphic in European populations and adjacent sites 4 bases upstream and downstream are reported.

Supplementary Table 15. Contingency table for X chromosome contamination estimates.

Sample		Test 1 Polymorphic sites	Test 1 Average of adjacent sites	Test 2 Polymorphic sites	Test 2 Average of adjacent sites
Kotias	**p**	1,773,694	1,849,832.125	214,334	222,645.625
	**s**	10,001	4,784.625	1,215	582.625
	**e**	0.006	0.003	0.006	0.003
Satsurblia	**p**	34,645	36,084.75	10,793	11,229.625
	**s**	155	76.875	44	24
	**e**	0.004	0.002	0.004	0.002
Bichon	**p**	948,648	979,946.500	139,533	143,799.625
	**s**	4,164	2,510.625	616	365.125
	**e**	0.004	0.003	0.004	0.003

"Test 1" and "test 2" were performed as in [11]. The table reports the number of primary alleles (p) and secondary alleles (s) in ancient male samples at X chromosome sites found to be polymorphic in European populations and the average of adjacent sites 4 bases upstream and downstream as well as the observed probability of error (e).

Supplementary Table 16. X chromosome contamination estimates.

Sample	Test	Contamination (%)	P-value
Kotias	1	0.99	$< 2.2 \times 10^{-16}$
	2	0.99	$< 2.2 \times 10^{-16}$
Satsurblia	1	0.56	5.22×10^{-8}
	2	0.58	0.0011
Bichon	1	0.54	$< 2.2 \times 10^{-16}$
	2	0.45	$< 2.2 \times 10^{-16}$

Estimates are based on “test 1” and “test 2”[11]. Associated p-values for contingency tables used in this analysis were calculated using Fisher’s exact test.

Supplementary Table 17. Molecular sex assignment of ancient samples.

Sample	R_y	Standard error	95% Confidence interval	Assignment
Kotias	0.0840	0.0001	0.0839-0.0842	Male
Satsurblia	0.0878	0.0002	0.0873-0.0882	Male
Bichon	0.0928	0.0001	0.0926-0.0930	Male

R_y, the ratio of the fraction of Y chromosome reads to the fraction of reads aligning to both sex chromosomes.

Supplementary Table 18. Mitochondrial haplogroups and haplotypes for Kotias, Satsurblia and Bichon.

Sample	Coverage	Haplogroup	Haplotype
**Kotias**	425x	H13c	73A, 146T, 152T, 195T, 247G, 769G, 825T, 1018G, 2706A, 2758G, 2885T, 3594C, 4104A, 4312C, 4769A, 7028C, 7146A, 7256C, **7521G**, 8206A, 8468C, 8655C, 8701A, 9540T, 10289G, 10398A, 10664C, 10688G, 10810T, 10873T, 10915T, 11719G, 11914G, 12705C, 13105A, 13276A, 13506C, 13650C, 14766C, 14872T, **16129G**, 16187C, 16189T, 16223C, 16230A, 16278C, 16311T, 16519T
**Satsurblia**	144x	K3	146T, 150T, 152T, **235G**, 247G, 560T, 769G, 825T, 1018G, 1097T, 1811G, 2758G, 2885T, 3480G, 3594C, 4104A, 4312C, 4769A, 6027T, 7146A, 7256C, 7498A, 7521G, 7657C, **8188G**, 8468C, 8655C, 8701A, 9055A, 9540T, 9698C, **9852T**, 10398A, 10550G, 10664C, 10688G, 10810T, 10873T, 10915T, 11299C, 11467G, 11914G, 12308G, 12372A, 12705C, 13105A, 13276A, 13506C, 13650C, 14167T, 14198A, 14212C, 14798C, 15924G, **16093C**, 16129G, 16148T, **16153A**, 16187C, 16189T, 16223C, 16224C, 16230A, 16278C
**Bichon**	314x	U5b1h	146T, 150T, 152T, 195T, 247G, **384G**, 769G, 825T, 1018G, 2758G, 2885T, 3197C, 3594C, 4104A, 4312C, **5656G,** 7028C, 7146A, 7256C, **7521G**, 7768G, 8468C, 8655C, 8701A, 9477A, 9540T, 10398A, 10664C, 10688G, 10810T, 10873T, 10915T, 11467G, 11914G, 12308G, 12372A, 12705C, 13105A, 13276A, 13506C, 13617C, 13650C, 14182C, 16129G, 16187C, 16223C, 16230A, 16270T, 16278C, 16311T, 16519T

Mutations reported here are with respect to the Reconstructed Sapiens Reference Sequence[12]. Mutations found in our samples which are present in the reported haplogroup are shown here unless marked in bold or underlined. Bold mutations are those expected for the prescribed haplogroup but not found in the sample. Underlined mutations are those present in our samples but not associated with the determined haplogroup.

Supplementary Table 19. Y chromosome haplogroups for ancient male samples.

Sample	Major haplogroup	Maximum likelihood haplogroup	Confidence haplogroup
Kotias	J	J	J
Satsurblia	J	J2a	J2
Bichon	I	I2a*	I2

Haplogroups were determined by Yfitter[13]. The "maximum likelihood haplogroup" is described by [13], as being the best guess haplogroup while the "confidence haplogroup" is described as the conservative guess haplogroup.

Supplementary Table 20. Genotypes for SNP panel used in 8-plex prediction system.

Gene	Marker	Genotype Bichon	Coverage Bichon	Genotype Kotias	Coverage Kotias	Genotype Satsurblia	Coverage Satsurblia
SLC45A2	rs16891982	CC	4C	CC	21C	CC*	-
IRF4	rs12203592	CC	13C	CC	19C	CC*	7C
SLC24A4	rs12896399	GT	5G,5T	GT	4G,9T	GG	-
OCA2	rs1545397	AA	8A	AA	5A	AA	-
HERC2	rs12913832	AG	6A,3G	AA	15A	AG^	2A,2G
SLC24A5	rs1426654	GG	25G	AA	13A	AA*	4A
MC1R	rs885479	GG	16G	GG	10G	-	-
ASIP	rs6119471	CC	10C	CC	24C	CC	-

* genotype supported by observed and imputed data

^ genotype supported by observed data

Observed genotypes are shown for Bichon and Kotias while imputed genotypes with probability greater than 0.85 are reported for Satsurblia unless otherwise marked. Coverage refers to coverage of observed high quality alleles with a depth ≥ 4x. Genotypes are reported with respect to the GRCh37 build of the human genome. Skin colour determination was inconclusive for all samples.

Supplementary Table 21. Genotypes for positions that define common haplotypes in the region surrounding the *SLC24A5* gene.

Marker	Ancestral allele	Selecte d allele	Genotype Bichon	Coverage Bichon	Genotype Kotias	Coverage Kotias	Genotype Satsurblia	Coverage Satsurblia
**rs1834640**	G	A	AA	8A	AA	15A	AA	-
**rs2675345**	G	A	GG	5G	AA	12A	AA	-
**rs2469592**	G	A	GG	11G	AA	25A	AA	-
**rs2470101**	C	T	CT	10C,1T	TT	16T	TT	-
rs938505	T	C	TT	9T	CT	6C,1T	CC	-
**rs2433354**	T	C	TT	9T	CC	9C,1T	CC	-
**rs2459391**	G	A	GG	18G	AA	26A	AA	-
**rs2433356**	A	G	AA	4A	GG	22G,1A	GG	-
**rs2675347**	G	A	GG	7G	AA	19A	AA	-
**rs2675348**	G	A	GG	9G	AA	19A	AA	-
**rs1426654**	G	A	GG	25G	AA	13A	AA*	4A
**rs2470102**	G	A	GG	9G	AA	12A	AA	-
rs16960631	A	G	AA	12A	AA	17A	AA	-
**rs2675349**	G	A	-	-	AA	6A	AA	-
**rs3817315**	T	C	TT	18T	CC	12C	CC	-
**rs7163587**	T	C	TT	11T	CC	19C	CC	-

* genotype supported by observed and imputed data

Observed genotypes are shown for Kotias and Bichon while imputed genotypes with probability greater than 0.85 are reported for Satsurblia. Coverage refers to coverage of observed high quality alleles with a depth ≥ 4x. Genotypes are reported with respect to the GRCh37 build of the human genome. Markers of the C11 haplogroup are shown in bold. This haplogroup is found in 97% of individuals with the derived rs1426654 variant[14]. Kotias and Satsurblia exhibit the the C11 haplogroup. Bichon does not.

Supplementary Table 22. Genotypes for the SNP panel used in the Hirisplex prediction system.

Gene	SNP	Genotype Bichon	Coverage Bichon	Genotype Kotias	Coverage Kotias	Genotype Satsurblia	Coverage Satsurblia
MC1R	N29insA	CC	8C	-	-	-	-
MC1R	rs11547464	GG	16G	GG	28G	GG	-
MC1R	rs885479	GG	16G	GG	10G	-	-
MC1R	rs1805008	CC	15C	CC	14C	CC	-
MC1R	rs1805005	GG	10G	GG	9C	GG	-
MC1R	rs1805006	CC	10C	CC	13C	CC	-
MC1R	rs1805007	CC	17C	CC	22C	CC	-
MCIR	rs1805009	GG	12G	GG	19G	GG	-
MC1R	Y1520CH	CC	15C	CC	19C	-	-
MC1R	rs2228479	GG	12G	GG	15G,2A	GG	-
MC1R	rs1110400	TT	14T	TT	16T	TT	-
SLC45A2	rs28777	CC	8C	CC	25C	CC	-
SLC45A2	rs16891982	CC	4C	CC	21C	CC	-
KITLG	rs12821256	TT	8T	TT	11T	-	-
EXOC2	rs4959270	CC	5C	CC	8C	AA	-
IRF4	rs12203592	CC	13C	CC	19C	CC*	7C
TYR	rs1042602	CC	11C	CC	19C	CC	-
OCA2	rs1800407	CC	11C	CC	8C	CC	-
SLC24A4	rs2402130	AA	18A	AA	24A	AA	-
HERC2	rs12913832	AG	6A,3G	AA	15A	AG^	2A,2G
PIGU/ASIP	rs2378249	AA	10A	AA	21A	GA	-
SLC24A4	rs12896399	GT	5G,5T	GT	4G,9T	GG	-
TYR	rs1393350	GG	14G	GG	14G	GG	-
TYRP1	rs683	CA	3C,2A	AA	4A	AA	-

* genotype supported by observed and imputed data

^ genotype supported by observed data

Observed genotypes are shown for Kotias and Bichon while imputed genotypes with a probability of greater than 0.85 are reported for Satsurblia unless otherwise marked. Coverage refers to coverage of observed high quality alleles with a depth ≥ 4x. Genotypes are reported with respect to the GRCh37 build of the human genome.

Supplementary Table 23. Hirisplex phenotypic predictions with accompanying associated probabilities for Bichon, Kotias and Satsurblia.

			Bichon	Kotias	Satsurblia
**Eye colour**	Associated probability	Blue	0.007	< 0.001	0.014
		Intermediate	0.027	0.003	0.033
		Brown	0.967	0.997	0.952
	**Prediction**		**Brown**	**Brown**	**Brown**
**Hair colour/shade**	Associated probability	Blonde hair	0.022	0.005	0.032
		Brown hair	0.275	0.164	0.259
		Red hair	< 0.001	< 0.001	< 0.001
		Black hair	0.704	0.831	0.709
		Light hair	0.030	0.006	0.060
		Dark hair	0.970	0.994	0.940
	**Prediction**		**Black/Dark**	**Black/Dark**	**Black/Dark**

Supplementary Note **1**

Archaeological context

Kotias Klde

Kotias Klde (Supplementary Fig. 6) is a rockshelter located at 707 m above sea level (a.s.l.) on a limestone plateau above the Kvirila River in western Georgia. Excavations took place from 2003-2006 and in 2010. The exposed stratigraphy showed four layers without attaining bedrock. Below we provide a description of the stratigraphy from top to bottom.

Layer A1 was 30 cm deep and contained the remains of a shallow pit-house, 2.5/3.0 m in diameter, which was dug into the underlying layers (A2 and B). Though there were a few pottery shards of Bronze Age and later periods, most of the material at the base of the layer belonged to the local Late Neolithic period, generally referred to as ‘Eneolithic’. These finds included a clay figurine[15] with nearby charcoal dated to 5,820 ± 40 uncal. BP (OS-90616).

Layer A2 contained a distinct lithic industry with transverse, trapezoid arrowheads. Special forms of denticulates known as "Lekalo" or "Kmlo" tools[16–18], often retouched on the ventral face, were represented in this assemblage. Flake scrapers, including the thumb-nail type, were also found. Blade cores and some production waste (debitage) reflect on-site blade reduction for secondary shaping of tools. A similar industry was reported in Neolithic sites of the region such as the Darkveti rockshelter, ca. 5 km away in the Kvirila river gorge[19], and Paluri, which is situated further away on the Enguri river[17]. Charcoal from this layer was dated to 7,430 ± 40 uncal. BP (OS-63263).

Layer A2 contained the grave of a complete skeleton from a young adult male (ca. 30-35 years old) whom we have called “Kotias”. The grave was dug from this layer into layer B. The skeleton was laid in supine position and stones were placed over certain parts of the skeleton which crushed the skull and covered the knees and lower limb bones. The skull was angled with the right side facing upwards. Both the right and left hands, which lay along the side of the skeleton, covered the groin area. A 15 cm long bone splinter was found in the chest cavity of the

specimen below the cervical vertebrae and above the clavicle on the right side. It cannot be affirmed, however, that this splinter was the cause of death. It appears that there were some pathological lesions on the right first rib. Other traumatic and degenerative pathological conditions were evident on the left calcaneus and talus. Attrition of the teeth was intense, especially when taking the relatively young age of the specimen into consideration. Two superimposed shallow hearths were exposed some 20 cm above the skeleton's legs probably either indicating the sealing of the grave or a later occupation. A sample from one of the hearths was dated to 8,370 ± 55 uncal. BP (RTT 5220). A tibia from the skeleton was directly dated to 8,665 ± 65 uncal. BP (RTT 5246) and a mandibular fragment to 8,745 ± 40 uncal. BP (OxA-28256). A combined calibrated plot provided an interval of 9,529-9,895 cal. BP (95.4%, 2 s.d.).

Layer B, interspersed with limestone fragments, was 50-60cm thick and subdivided into three sub-layers (B1-B3) all of which contained evidence of Mesolithic industry and animal bones. A total of nine radiocarbon dates from animal bones and charcoal of this layer yielded a calibrated interval range of 10,300-13,000 cal. BP. Artefacts were made of flint, radiolarite, rare crystal rock, and obsidian (the latter was obtained from exposures some 80 km away). The main tool groups were backed and retouched bladelets. Numerous scalene triangles shaped by bi-polar retouch from blades and bladelets define the Mesolithic industry[20]. In addition, a few bone tools were recovered, including a handle made of a red deer antler hollowed for hafting a tool, as well as several point tips.

Layer C, consisting of yellow, compact clay/loamy sediment, was exposed to a very limited extent. The lithic industry was of Upper Palaeolithic age characterized by blade production and the presence of end-scrapers and burins. A total of 9 radiocarbon dates from animal bones and charcoal of this layer yielded a calibrated interval range of 22,200-23,600 cal. BP. The time gap between these two occupations (Layers B and C) indicates that the cave was not inhabited during the Last Glacial Maximum (LGM) and for quite some time after the LGM until the Younger Dryas and the first millennium of the Holocene.

Satsurblia

Satsurblia cave in western Georgia (Supplementary Fig. 6) was discovered in 1975 by A. N. Kalandadze[21] who excavated it sporadically during 1976, 1985–88 and 1990-1993[22]. A second series of excavations was conducted during 2008–2010, directed by Tengiz Meshveliani.

Further excavations in Satsurblia (2012-2013), directed by Tengiz Meshveliani and Ron Pinhasi, were conducted in two areas. Area A was situated in the north-western part of the cave, near the entrance (squares R−T 20−24) and Area B was to the rear of the cave (squares T−Z 4−7), adjacent to a trench excavated by K. Kalandadze in the 1980s. Both areas revealed stratigraphic sequences comprising Pleistocene (Upper Palaeolithic) and Holocene (Eneolithic and later) deposits. Excavations in 2012 and 2013 focused on Areas A and B with both yielding in situ Upper Palaeolithic layers that were extremely rich in finds (a circular fireplace, large quantities of charcoal and brunt bones, lithics, bone tools, shell ornaments, yellow ochre) and which continued to unknown depth. The Upper Palaeolithic sequence of Area A was divided into two main units: A/I and A/II. A/II contained a sequence of living surfaces which were dated (surface II and III) to 17,000-18,000 cal. BP and as such are the first well-dated evidence for human occupation in the southern Caucasus at the end of the LGM. This fills a gap in the Upper Palaeolithic sequence, namely that between Unit C and Unit B in Dzudzuana Cave (western Georgia), dated to 27,000-24,000 cal. BP and 16,000 cal. BP - 13,200 cal. BP respectively.

Preliminary analyses of lithics from Satsurblia cave reveal a cultural variant which resembles Eastern Epi-Gravettian industries, dominated by bladelets and including varieties of microgravette points and bigger gravette points. A rectangular tool type was novel to excavations from the region and differed from the geometric trapezoid-rectangle tools of the proceeding Mesolithic cultures in size, shape and retouch[23] Direct AMS dating of human remains from Area B yielded the first securely dated Upper Palaeolithic human remains from the Caucasus. “Satsurblia” was sampled from a right temporal bone which was recovered in 2013 from square Y5 and dated to 11,415 ± 50 uncal. BP (OxA-34632). A combined calibrated plot provided an interval of 13,132-13,380 cal. BP (95.4%, 2 s.d.).

Grotte du Bichon

The small cave "Grotte du Bichon" is situated in the Swiss Jura Mountains, a few kilometres north of the city of La Chaux-de-Fonds (canton Neuchâtel), at an altitude of 845 m a.s.l. (Supplementary Fig. 6). During its first excavation, undertaken by speleologists in 1956, bones of a young man were discovered intermingled with the remains of a female brown bear (*Ursus arctos*) and nine flint projectile points, apparently stemming from the hunter's weapons.

Both skeletons as well as the flints were located about 15 m from the entrance, at a recess of the cave, and were associated with charcoal concentrations but with no other archaeological material. Although a hunting accident was already envisioned at that time, without further indications the remains of the bear were considered not to be related to the human skeleton and therefore stored at the natural history museum of La Chaux-de-Fonds, while the human bones and the flints were kept in the archaeology museum of Neuchâtel (Laténium).

Only in 1991, during re-examination of the animal bone material, did archaeozoologist Philippe Morel discover an impact trace and two flint chips (probably a broken tip of an arrowhead) in a cervical vertebra of the bear, thus establishing a clear connection between the animal and the man[24]. This discovery prompted new excavations that were carried out between 1991 and 1995. During these new investigations, using modern excavation techniques (including water sieving), all of the missing long bones from the two skeletons were recovered, together with some more flint artefacts. The small lithic assemblage now contained 10 backed points, 16 backed bladelets and one retouched blade fragment, characteristic of final (Azilian) Palaeolithic industries. It seems that the cave was never used as a camp site as unretouched debitage products were not recovered. Four radiocarbon measurements performed on the bones from the bear and the man (two on each) and eight dates obtained from charcoal, from willow (*Salix sp.*) and from pine (*Pinus sylvestis*) ranged from 10,950 to 11,760 uncal. BP[25]. A new direct date on the human skeleton 11,855 ± 50 uncal. BP (OxA-27763) or 13,560- 13,770 cal. BP (95.4% CI, previously unpublished) is in agreement with the dates of the charcoal and pine.

The human skeleton was determined to be of a young male, 20-23 years of age, of the Cro-magnon type. According to the cranio-facial architecture, it was characterized by classical cranio-facial disharmony, i.e. a relatively long skull associated with a low face and sub-rectangular eye-sockets, which are quite typical of the time period. The young man weighed a little over 60 kg and stood 1.64 m tall. Although of a relatively slender build, muscle attachments showed him to have been a strong runner and well adapted to mountainous terrain[26]. His upper limbs show a high degree of asymmetry, indicative of preferential use of the right arm[27]. Isotopic studies of carbon and nitrogen fractionations indicated a largely meat based terrestrial diet[28].

Supplementary Note 2

Authenticity of results

Estimation of molecular damage and sequence length

Ancient DNA molecules are subject to post-mortem degradation typified by short sequence length and an over-representation of nucleotide misincorporation sites at the ends of reads[29,30]. Using a subsample of 500,000 reads per sample, which had been processed as described in the methods section with the SeqTK step omitted, we examined the sequence length distribution of our reads (Supplementary Fig. 7) and patterns of molecular damage using mapDamage 2.0[31] (Supplementary Fig. 8). Only bases with a minimum quality of 30 were considered when running mapDamage.

Endogenous DNA sequences from ancient samples tend to have an average sequence length of less than 100 bp[32] and for Kotias and Satsurblia a peak in DNA sequence length was observed at 47 bp and 48 bp respectively, with a second peak at 100bp (Supplementary Fig. 7A&B). As 100 bp sequencing was performed, all sequences greater than this length are truncated, inflating the count of 100 bp reads. The peaks at < 50 bp are more likely an accurate reflection of the modal sequence length of the molecules. Bichon shows a large peak at 100 bp with a longer, flatter distribution of reads than that of the Georgian samples (Supplementary Fig. 7C). A different extraction protocol was used for Bichon[33] than for Satsurblia and Kotias[2]. Different extraction protocols can result in distinctive sequence length distributions for ancient next-generation sequencing data[34] and this, along with variation in DNA preservation, may have contributed to the observed differences in the length of sequences between the Caucasus hunter-gatherers (CHG) samples and Bichon.

For each sample a clear increase in C to T and G to A transitions can be seen at the 5' and 3' ends of molecules respectively (Supplementary Fig. 8), a typical hallmark of aDNA[29,30]. Misincorporation frequencies at the start and end of reads (>20% for the Georgian samples and >7% for Bichon) are consistent with levels present in other similarly aged specimens[1,2,6,8]. Bichon may have less damage at the 5' ends of molecules (the 3' ends were not always

completely sequenced) than CHG because DNA fragments retrieved from Bichon tended to be longer (Supplementary Fig. 7) and thus better preserved.

Mitochondrial DNA contamination

Mitochondrial DNA contamination was evaluated by assessing the proportion of secondary (non-consensus) bases at haplogroup defining positions in our ancient samples[2,38]. Haplogroup defining positions determined using HAPLOFIND[42] were called in our samples using GATK Pileup[36] and filtered to remove bases with a quality < 30. The contamination rate (“C + MD”) was determined by calculating the secondary base count as a fraction of the total base count at all haplogroup-defining positions[2,35]. Because of the possible influence of residual molecular damage on estimates, this was also repeated omitting sites where the secondary base could be explained by deamination (“C”)[2,35]. We found low contamination rates of ⋝ 0.62% for all samples (Supplementary Table 13).

X chromosome contamination

As all our samples were male (Supplementary Table 17) we were able to assess the level of X chromosome contamination in our samples as described in [2] which was based on [11]. The X chromosome is found in single copy in males and therefore two or more different alleles found at a given site along this chromosome may be due to contamination, damage, sequence error or mismapping. Assuming a similar background site error rate, it is expected that contamination will be more conspicuous at polymorphic sites than at neighbouring monomorphic sites due to the increased propensity for allelic diversity at these sites in contaminating populations. We examined discordance in the rate of heterozygous calls between known polymorphic sites on the non-pseudoautosomal region of the X chromosome reported in the 1,000 Genomes Project[36] and their adjacent sites. Genotypes in the 1,000 Genomes dataset and ancient samples were called and filtered according to [2] with a minimum sequence depth of 5 required for genotypes called in Kotias and Bichon and 3 for loci called in Satsurblia (a lower threshold was used for Satsurblia due its comparatively lower coverage). Two tests, which employ a maximum likelihood based approach, were performed to evaluate the rate of contamination. “Test 1” uses all high quality reads provided per sample while “test 2” removes the assumption of independent error rates by only sampling a single read per site (Supplementary Tables 14 & 15)[11]. A very low

contamination rate of 0.99% (p-value $< 2.2 \times 10^{-16}$) was found for Kotias with similarly low levels of 0.56-0.58% (p-value = 0.001) for Satsurblia and 0.45-0.54% (p-value $< 2.2 \times 10^{-16}$) for Bichon (Supplementary Table 16).

Supplementary Note 3

Kotias and Satsurblia form a clade with respect to other ancient samples while Bichon shares close affinity to other western hunter-gatherers

D-statistics of the form *D*(Yoruba, *OA*; Satsurblia, Kotias) and *D*(Yoruba, *OA*; Bichon, *WHG*) were used to assess whether the pairs of samples (Kotias, Satsurblia) and (Bichon, *WHG*) are compatible with forming a clade in an unrooted tree with respect to an African outgroup and other ancient samples (OA). For the test *D*(Yoruba, *OA*; Satsurblia, Kotias) we found most statistics to have non-significant (zero) values (Supplementary Table 2) which supports Kotias and Satsurblia forming a clade to the exclusion of other branches of ancient ancestry. The only population for which a positive value was observed was the Sintasha Bronze Age culture. These Uralic people are genetically similar to Corded Ware populations[10] and this result could be explained by the temporally closer Kotias representing a better donor for CHG ancestry than the older Satsurblia for this population. These *D*-statistics confirm inferences from PCA and ADMIXTURE that Kotias and Satsurblia are genetically distinct from other broadly contemporaneous ancient genomes. When we performed the tests *D*(Yoruba, Satsurblia; *OA*, Kotias) and *D*(Yoruba, Kotias; *OA*, Satsurblia) we found positive values of 0.07-0.16 with corresponding Z-scores of 9.66-24.18. This shows that there is enough power to detect signals of admixture using this dataset and that the zero values found above are not due to paucity of data.

The test *D*(Yoruba, OA; Bichon, WHG) (where WHG were represented by the highest coverage WHG genomes, Loschbour[1] and La Braña[6]) resulted in insignificant values for 95% of tests consistent with Bichon having more recent shared ancestry with WHG than with most other ancient lineages (Supplementary Table 3). We consistently found zero-values when the OA involved was an eastern hunter-gatherer (EHG), a CHG or a Pleistocene hunter-gatherer showing that Bichon forms a clade with WHG to the exclusion of these other hunter-gatherer groups. We did not always find zero-values however when we let OA be a Scandinavian hunter-gatherer (SHG; Supplementary Table 3). WHG are proposed to be part of a hunter-gatherer metapopulation, which also encompasses SHG and EHG, that ranged over northern Europe from as far west as Spain to as far east as Russia[7]. These three hunter-gatherer groups cannot be related by a simple tree as there are signals of admixture between these groups[7]. This

explains why Bichon does not always form a clade with other WHG to the exclusion of SHG. When we inverted the statistics and evaluated *D*(Yoruba, Bichon; *OA*, WHG) and *D*(Yoruba, WHG; *OA*, Bichon) we consistently found statistically significant values (Z >3). This shows that admixture can be detected for the genotype coverage found in this dataset. We also found similar results when we let the Hungarian sample KO1[2] represent WHG. It is interesting to note that Bichon, as well as other WHG, form a clade with both MA1 and EHG to the exclusion of CHG (Supplementary Table 6). This suggests the Ancient North Eurasian (ANE) ancestry and WHG ancestry may have shallower roots and diverged subsequent to splitting from CHG (Supplementary Fig. 2). This is consistent with ADMIXTURE analysis and the geographic range of these groups - CHG were separated from these North Eurasian hunter-gatherers by the Caucasus mountain range.

We also explored the relationships between ancient samples by performing outgroup f_3-statistics of the form $f_3(X, OA;$ Yoruba) where we let X be Kotias, Satsurblia and Bichon in turn and OA be all other ancient groups in the dataset (Supplementary Fig. 1). These statistics are informative as their magnitude is proportional to the amount of shared genetic history between the ancient individuals (X and OA) since they diverged from an African (in this case Yoruban) outgroup.

We found that CHG share the most drift with each other and the least drift with the Pleistocene sample Ust'-Ishim (Supplementary Fig. 1A&B). Other ancient samples share an intermediate amount of drift with no obvious pattern to the distribution of allele sharing. Bichon shares the most genetic drift with other western hunter-gatherers, followed by Scandinavian and eastern hunter-gatherers (Supplementary Fig. 1C). The fact that Bichon is closest to other WHG, and not equally close to SHG and EHG, suggests that there may have already been sub-structure between these hunter-gatherer groups 13,700 years ago when Bichon was alive.

Supplementary Note 4

Caucasus hunter-gatherers and early farmers are sister groups with an earlier divergence for western hunter-gatherers

To explore the topology between CHG, WHG and early farmers (EF) we used available high coverage data and performed f_3-statistics (see methods), attempting all possible triplet combinations for these three groups (Figure 2A; Supplementary Table 4). When we did this we presumed that two samples form a clade and the other sample is the outgroup to this clade. For the correct topology we would expect $f_3 > 0$, as the two correctly grouped samples will have shared drift since they diverged from the outgroup. For incorrect topologies we would expect $f_3 = 0$ as the incorrectly grouped samples will not have shared drift exclusive to themselves. We found that f_3(*WHG, CHG*; *EF*) tended to equal zero and gave the smallest values of all our tests. This makes it unlikely that WHG and CHG are sister groups to the exclusion of EF. The largest values were found for f_3(*CHG, EF*; *WHG*) (Z > 14.4) suggesting that CHG and EF form a clade to the exclusion of WHG. We did however also find positive statistics for the test f_3(*WHG, EF*; *CHG*) (Z > 8.5) but these were not as significant as for the former topology. WHG introgression into EF has been proposed previously [1,2,4,5] and positive statistics for f_3(*WHG, EF; CHG*) could be a function of this admixture (admixture is also suggested by *D*-statistics of the form *D*(Yoruba, WHG; CHG, EF) (Supplementary Table 8) and ADMIXTURE analysis (Figure 1B)). As the signal for EF and CHG forming a clade is much stronger than for the other two topologies we consider the most parsimonious scenario to be that farmers and CHG are sister groups that diverged from each other after splitting from WHG.

Unfortunately we did not have a high coverage diploid sample representing ANE to include in these analyses. Analyses using *D*-statistics (Supplementary Table 6) revealed however that ANE and WHG group together to the exclusion of CHG. It therefore seems likely that an ancient south (Neolithic farmers and CHG) divergence from the ancient North (WHG and ANE) was the earliest split for these groups. This is shown in Supplementary Fig. 2 which extends the model proposed in [1] to include CHG. To fit this proposed model CHG and EF should form a clade to the exclusion of Eastern non-Africans which is indeed supported by zero values for *D*(Yoruba, *eastern non-African*, *CHG*, *EF*) (Supplementary Table 8). CHG and EF also form a clade to the exclusion of ANE as represented by MA1 (Supplementary Table 8).

Supplementary Note 5

Dating the split among Caucasus hunter-gatherers, western hunter-gatherers and early farmers

We used G-PhoCS[54] to reconstruct the joint demographic history of western and Caucasus hunter -gatherers (WHG and CHG respectively) and early farmers (EF). This analysis requires (1) the topology of the underlying population tree; (2) sequence data from short, homologous windows; and (3) specified directional gene flow between branches (migration bands).

Topology of population tree

G-PhoCS represents the demographic history of a collection of samples by a (binary) tree in which each branch is a population, with each sample belonging to a different leaf branch and interior branches corresponding to ancestral populations. To find the most likely topology of this tree, we used f_3 analysis to determine the most likely ordering of the population splits (see Figure 2B for a graphical representation). For the G-PhoCS analyses, we considered both a tree with only the ancient genomes, and a tree with an African San Pygmy[55] as the outgroup.

Genome-wide windows of high sample coverage for demographic analyses

Since this analysis requires sequence data from all genomes in short (1 kilobase (kb)) homologous windows, we chose high-coverage genomes to represent each group (Bichon and Loschbour to represent WHG, Kotias to represent CHG, and either Stuttgart or NE1 to represent EF). In addition, we used a high-quality San Pygmy genome[55] as an outgroup. To find the best set of windows, we first generated all-sites coverage information for chromosomes 1 to 22, restricted to regions classified as "neutral" according to the filters in[54] (using UCSC liftOver tool to translate coordinates from hg18 to hg19), and extracted the depth information using a program written in C, again filtering for sites with read depth between 10 and 35 (we avoid sites with very low and very high coverage because alignment and genotyping is problematic (for more details see [37]):

```
samtools mpileup -C50 -uDI -f <reference.fa>  -r <chromosome> \
-l <bedfile with accepted regions> <bamfiles> | bcftools view -gc – \
| get_depth_intervals minCover=10 maxCover=35
interval_file=<chromosome>
```

We then scanned each chromosome for good windows, using a simple heuristic to maximise the sample coverage. We start by finding the first 1 kb window with at least 80% coverage. We then search locally for a window within the next 10 kb for the 1 kb window with the highest coverage. Finally, we jump 5 kb forward from the chosen location and repeat the process until we reach the end of the chromosome. For the whole genome, this search yielded a total of 152,883 high-quality windows. We then used SAMtools/BCFtools[56] (using flags as above) and custom programs written in C and MATLAB to extract genotypes for the windows and converted the genotypes into fasta files for G-PhoCS. To deal with DNA damage in ancient samples, we "in vitro" deaminated all our sequences, as already done for previous analyses of aDNA[57].

Directional gene flow between branches

Because our WHG samples predate the arrival of farming to central and northern Europe[58], any gene flow creating shared drift between EF and WHG must be from WHG to EF. Ideally, we would like our model to only allow gene flow between WHG and EF after the arrival of farming to the WHG locations. However, G-PhoCS requires migration to start or stop at time where populations split. Fortunately, our analysis puts the split between the two WHG, Bichon and Loschbour, at around 14k years ago, just a few thousand years prior to farming. We therefore allow gene flow between WHG and EF only after this split. Because Loschbour is temporally and geographically closer than Bichon to the EF, we allow only gene flow from Loschbour to the EF.

G-PhoCS set up

Gamma distributed priors were used for all observables. The shape parameter α was set to an intermediate value of 1 for both population sizes and split times, to obtain a mean to standard deviation ratio of unity and allow sufficient exploration of the parameter space without an overly

long convergence time. The rate parameter β was set to result in means in the correct range, based on initial exploratory runs with a variety of starting values (running two MCMC chains of 1,000,000 steps for each set of starting values, which is enough to get an order of magnitude estimate of variables of interest; see below for details of how chains were set up). For θ, the effective population sizes, we set β=2,500 for the San (i.e. for θ_{San}), and β=10,000 for all other populations, corresponding to mean effective population sizes of 26,667 and 6,667, respectively. The rate parameters were β=1,000,000 (mean split time of 6,667 years) for the Bichon-Loschbour and Stuttgart-Kotias splits, β=250,000 (mean split time of 26,667 years) for the (Bichon-Loschbour)-(Stuttgart-Kotias split) and β=30,000 (mean split time of 222,222 years) for the San and ancient genomes.

With regards to migration from Loschbour to Stuttgart, we explored three different settings: no migration and migrations bands with either a strong or a weak prior. In the case of a weak prior, the shape parameter was set to 0.002 and the mean to 200, to allow exploration of the whole space. With such a broad distribution, the value of the mean hardly influenced our results, based on exploratory runs. As for the strong prior, the shape parameter was set to 1 and the mean to 20,000, corresponding to ~18% of the genealogies sampled as Stuttgart actually originating from Loschbour, with a Loschbour-Bichon split time of 6,666 years (the prior mean). Migration had a negligible effect on split times. The only exception was the split between EF and Caucasus Hunter Gatherers, which was approximately 4-10 thousand year younger without migration; however, the confidence interval for this split was similar, and very broad, for levels of migration, suggesting that this split is difficult to date with the data we have. In this supplementary, we present the results with the strong prior, since previous studies[1,7,46] have pointed to mixing between hunter-gatherers and early farmers. We also explored models with migration going in the opposite direction or bidirectional, but this did not affect the results (as already noted in the original paper describing G-Phocs[54], the direction of migration tends not to be captured by this method).

We used G-PhoCS's automatic feature to set step sizes (finetune parameters) of the Markov chain for each parameter, which aims for intermediate acceptance ratios. During this procedure, the first 10,000 steps were used to find appropriate step sizes, by updating the parameters every 100 MCMC steps and performing 100 updates. After the step sizes were set, 3,000,000

MCMC steps were performed, with the first 100,000 steps discarded as burn-in. After observing traces of observables, we found that our demographic parameters of interest converged well before the end of the burn-in period. We started two independent chains for each setting in order to assess appropriate mixing of the chain, and observed no problems in any setting.

Converting dates from ancient genomes

G-PhoCS assumes samples to be contemporaneous. The ages of our ancient genomes all fell within a range of ~6k years (~7 kya for the youngest, EF, to ~13 kya for the oldest, Bichon). This discrepancy is relatively small compared to the ages of the splits of interest, and will not affect estimates in a qualitative way (especially given the size of the confidence interval of this type of analysis). To convert split times for a given node as computed by G-PhoCS into calendar dates, we added the mean of the ages of the samples that defined that node. The only modern genome is the San, which is only used as an outgroup; as such, the age of that split between the San and the ancient genomes is not of interest (and given how old that split is, a difference of 10k years in age of the genomes has negligible consequences on the estimates).

Split times estimates from G-PhoCS have to be converted into calendar years based on a mutation rate. Recent work on the high quality genome from Ust'Ishim[3] provides a mutation rate calibrated on ancient DNA, (0.5×10^{-9} per site per year) which is also in line with estimates from high quality modern genomes[59]. We converted this mutation rate into an appropriate substitution rate for our *in vitro* deaminated sequences.

G-PhoCS Results

If we consider the model with Stuttgart to represent EF, and San as an outgroup, we find that the split between WHG and the population ancestral to CHG and EF is dated at around ~46 kya, implying an early divergence at the time of, or shortly after, the colonisation of Europe. On the WHG branch, the split between Bichon and Loschbour is dated to ~18 kya (just older than the age of Bichon), implying continuity in western Europe. The split between CHG and EF is dated at ~24 kya, thus suggesting a possible link with the LGM, although the broad confidence intervals require some caution with this interpretation.

Our conclusions are qualitatively similar for other models, irrespective of which genome (Stuttgart or NE1) was used to represent EF, nor whether we used an outgroup (San) or not. In the main text and in Figure 2B, we report the dates from the model including Stuttgart and San, but details of the other models are available in Supplementary Table 5.

Supplementary Note 6

Phenotypes of interest

To get a picture of the phenotypic characteristics of our samples, we examined genes which have been associated with particular phenotypes in modern populations, including some loci which have been subject to selection in European populations. To investigate skin tone in our samples we began by using the 8-plex prediction model[66], a tool developed for forensic applications. We found the skin colour results for Kotias, Satsurblia and Bichon to be inconclusive (Supplementary Table 20). To explore further we looked at genotypes in two pigmentation genes proposed to have been strongly selected in the ancestors of modern Europeans, namely *SLC45A2* and *SLC24A5*[14,67–71]. Skin colour tends to get progressively paler with increasing distance from the equator[72] and this pattern is thought be the result of natural selection. In higher latitudes, with restricted ultraviolet radiation exposure, lighter skin colour confers a selective advantage as it allows increased dermal vitamin D synthesis[73,74]. Selected SNPs in the *SLC45A2* and *SLC24A5* genes (rs16891982 and rs1426654 respectively) contribute to lightening of skin and are almost fixed in modern Europeans[14,67–71]. We found that Kotias and Satsurblia have the ancestral version of the *SLC45A2* (rs16891982) variant but both CHG have the selected version of the *SLC24A5* (rs1426654) gene (Supplementary Table 21) encompassed by the most commonly associated haplotype (C11) found in modern populations[14]. Bichon on the other hand has the ancestral version of both genes suggesting that our Caucasus hunter-gatherers may have had lighter skin than our western hunter-gatherer, Bichon (Supplementary Table 20).

We used the Hirisplex online tool to predict hair and eye colour for our samples[75]. We found it most likely that Bichon, Kotias and Satsurblia had dark/black hair and brown eyes (Supplementary Tables 22 & 23). It is unlikely that any of our samples were able to drink milk into adulthood as all samples had the ancestral genotype at two positions (rs4988235 and rs182549) upstream of the *LCT* locus where the derived genotype is associated in Europeans with the ability to process lactose. The ability to digest milk is thought to have been driven to high frequencies in Europe subsequent to the introduction of farming[2,76].

Supplementary Note 7

Runs of homozygosity

To gain an insight into past population structure we examined runs of homozygosity (ROH) in our ancient samples. ROH occur when identical extended regions of the genome are inherited from both parents and their distribution can be informative about past population demography[2,60–62]. Long homozygous genomic stretches provide evidence for recent endogamy because recombination has not acted to break down these long tracts which are identical by descent. In contrast short runs can be indicative of an ancient population bottleneck. After such a constrictive event a population will experience a period of increased inbreeding creating long homozygous haplotypes but these segments can be broken up by recombination over time as the population expands, creating short homozygous runs.

Examination of ROH requires dense diploid genotypes. We used imputation to maximise the information content of our most ancient sample, Satsurblia, which was sequenced to 1.44x. Imputation allows the inference of missing genotypes by comparing surrounding haplotypes in the sample to those found in a phased reference panel and has been shown to be a valid method for leveraging palaeogenomic data[2]. We were concerned that the haplotypes present in Satsurblia may not be well represented by haplogroups in our modern dataset. To test if CHG genotypes could be accurately imputed we down-sampled our high coverage genome Kotias to ~1x and compared 546,625 imputed genotypes which overlapped with our confidently called high coverage genotypes for the filtered Human Origins dataset (Supplementary Fig. 10). When we imposed a genotype probability of 0.99 we found that 85% of loci were retained and of those 99.41% (97.91% of heterozygotes) matched our high coverage calls (Supplementary Fig. 8). This high concordance rate supports the use of imputed CHG data in our analyses.

ROH analysis was carried out as described in the methods section. When we plotted short ROH (<1.6 Mb) against long ROH (≥ 1.6 Mb) (Figure 3A)[2,61] we found that our three hunter-gatherer samples, Satsurblia, Kotias and Bichon locate with other hunter-gatherer samples in a region of the plot with a relative excess of both short and long ROH compared to Neolithic farmers. These

short runs suggest an ancestrally restricted population size for CHG as well as WHG which could reflect the effect of a reduced population size in glacial refugia. This contrasts with the relatively low frequency ROH found for Neolithic samples, perhaps because the ancestors of these people resided in a location further south with a more moderate climate during the LGM, permissive of a larger effective population size. Longer runs of ROH found in hunter-gatherer samples are compatible with more recent consanguinity in their family lines than that experienced by Neolithic farmers.

Kotias, Bichon and Loschbour all overlap with individuals from America with the former two samples also overlapping Oceanic individuals. Both American and Oceanic populations have experienced a population bottleneck during their histories[60]. Of all the ancient samples Satsurblia has the most long ROH and lies closest to the Onge, indigenous people from the Andaman islands. This island population has experienced long term isolation resulting in a small ancestral population size[63] and recent population reduction after colonisation by the British in 1858[64].

We also placed our ROH into size bins[60] (Figure 2B) and found Neolithic farmers to have a ROH distribution that follows a similar pattern to modern Eurasian groups. In contrast hunter-gatherers had a relative excess of ROH <4 Mb in size, a signature of a small ancestral population size. Compared to other hunter-gatherer samples, Satsurblia has an excess of long ROH 4-16 Mb in size suggestive of a more recent interbreeding event in the family history of this individual.

Supplementary Note 8

Uniparental haplogroups

Mitochondrial haplogroups

Kotias (425x coverage of the mitochondria) was assigned to haplogroup H13c (see methods). Mitochondrial haplogroup H, the most prevalent and diverse haplogroup found in west Eurasia, peaks in frequency in western Europe, accounting for more than 40% of total mtDNA diversity with a decreasing yet still appreciable frequency towards the Near East, the Caucasus and Central Asia (10-30%)[38]. Coalescence age estimates are considerably older for H in the Near East (23-28 kya) than in Europe (19-21 kya) and it has been proposed that H may have evolved in the southern Caucasus and northern part of the Near East where the most ancient clades of H are present[38–40]. Sub-haplogroup H13 is most common in the Near East and Caucasus reaching highest frequencies in Georgia and Daghestan[39]. Interestingly this sub-haplogroup has been found in individuals from the Late Neolithic Bell Beaker culture in Germany and the Early Bronze Age Yamnaya culture from the Pontic Steppe[7]. Individuals from these cultures are proposed to have a component of Near Eastern ancestry distinct from that of Early European farmers[7]. H13 has an estimated coalescence time of 20-24 kya (17-24 kya for H13c) thus placing the origin of this subclade during the LGM or even before[39].

Satsurblia (144x) was assigned to haplogroup K3. Satsurblia lacked 5 of the 11 mutations associated with the K3 haplogroup (Supplementary Table 18)[41,42]. These "missing" mutations (all sites had a minimum of 119x coverage) are all on the branch leading from K to K3 suggesting that the haplotype of Satsurblia could represent an early manifestation of the K3 haplogroup. Haplogroup K is found at about 11% frequency in Georgia today with similarly high level found in the Near East[43]. Its average European frequency is 5.6%[43] however it has been a found at higher frequencies in Early European Neolithic farmers and its diffusion in Europe has been associated with the Neolithic transition[44]. Haplogroup K was found to be the predominant haplogroup among samples from Pre-Pottery Neolithic B sites in Syria[45]. This culture is thought to represent one of the original Near Eastern Neolithic communities.

Bichon (314x) belongs to haplogroup U5b1h. The U branch, especially haplogroup U5, has been

found to be a dominant mitochondrial haplogroup among European hunter-gatherer communities[1,6–8,35,46,47].

Y-chromosomal haplogroups

Both Georgian hunter-gatherer samples were assigned to haplogroup J with Kotias belonging to the subhaplogroup J2a (see methods). Haplogroup J is estimated to have arisen 31.7 kya in the Middle East and is widely distributed in Eurasia, the Middle East and North Africa[48,49]. Patterns of haplogroup frequency are consistent with an expansion from the Middle East towards Europe which has been suggested to have accompanied the Neolithic transition in Europe[48,50,51]. In a study exploring J haplogroups in 445 individuals from Eurasia, J2a was found at highest frequency in Georgia and Iraq[48]. It is intriguing that both the mitochondrial and Y chromosome haplogroups of our ancient Georgian samples have been associated with the Neolithization of Europe. This tentatively suggests a genetic link between Georgian hunter-gatherers and early European migrants from the Near East.

Bichon belongs to Y haplogroup I2a (see methods). Haplogroup I has been found at high frequencies in Europe but is virtually absent elsewhere[52]. This haplogroup is suggested to have a European pre-LGM origin[53] and has been found in ancient samples with hunter-gatherer backgrounds from central and northern Europe[1,2,7,8].

Supplementary Note 9

ADMIXTURE analysis

The admixture proportions are shown on Supplementary Fig. 5 for all samples and in Figure 1 for ancient individuals at *K*=17 (the minimal cross validation error; Supplementary Fig. 9). In Supplementary Fig. 5, samples are hierarchically clustered by region (as on the PCA plot) and population. For better visibility, ancient samples are positioned on the left side of the figure and represented as bars with a width corresponding to five individuals. There are no clear outliers in any population, suggesting that they were well-defined and that the number of SNPs was sufficient to correctly define the clusters, even after LD-based pruning.

The cluster membership of published modern and ancient samples is similar to previous analyses[7]. Modern individuals harbour components as expected from their location and history, and ancient samples have components similar to those seen in [7] and other studies. European hunter-gatherers from the Mesolithic form a distinct component ("light blue"), which is also present in most Europeans and many populations from Western Asia. Early European farmers as well as modern European and West Asian groups additionally harbour a component dominant in the Middle East ("magenta"), appearing at *K*=9, in agreement with a Middle Eastern source of early Neolithic farmers. European groups from the late Neolithic onwards and many West Asian groups also possess a component prevalent in South Asia, as soon as this appears at *K*=7. This component is at first the same one that is prevalent in India ("dark purple"), but is later replaced by the Caucasus-related component ("lime green").

Bichon, our Upper Palaeolithic hunter-gatherer from Switzerland, harbours mainly the European Mesolithic hunter-gatherer component, in agreement with our PCA analysis while the Caucasus hunter-gatherers look unlike any other modern or ancient group. From *K*=9 to *K*=14, when both the South Asian "dark purple" and the Near Eastern "magenta" are present, they mainly consist of those two components, with a small fraction of Western hunter-gatherer related ancestry. Modern populations from the Caucasus and West Asia harbour the same components, but with an increased fraction of the Near Eastern "magenta" component. From *K*=15 onwards, a new cluster nearly completely describing CHG ("lime green") appears. Out of the two CHG samples, the older Satsurblia is fully assigned to the "lime green" cluster, whereas the later sample Kotias

also features minor (<10%) Near Eastern ancestry. This new CHG-related component also appears in modern populations in west Eurasia, but no modern population belongs purely to this cluster. Even modern populations from the Caucasus continue to harbour a large amount (close to 50%) of the Near Eastern “magenta” component.

Both Kotias and Satsurblia show a certain similarity to the early European Farmers (EF) and the Yamnaya, in that they feature both the Middle Eastern component of the EF and the South Asian of the Yamnaya. However, in contrast to EF and the Yamnaya and in agreement with a deep CHG-WHG split, they only harbour a minor proportion (<10%) of the European hunter-gatherer ancestry. Also from K=15 onwards, the new CHG-related component replaces the South Asian “dark purple” and reduces the Near Eastern “magenta” components in the Yamnaya and all Neolithic and later European populations.

The components characteristic of Native Americans populations (“dark green”, “light brown”, “dark gray” and “dark blue”) are also worth noting. These components are present in MA1 and eastern hunter-gatherers, in agreement with previous studies[5,7], but is at very low levels in the Yamnaya and virtually absent from our samples from the Caucasus. These components may point to the presence of the Ancient North Eurasian ancestry of both native Americans and eastern hunter-gatherers. Their absence in Caucasus hunter-gatherers is in agreement with their southern geographical position and their separation from northern Eurasia by the Caucasus mountain range.

Supplementary Note 10

Processing of published ancient data

Hungarian ancient data

MapDamage 2.0[31] was used to rescale selected high coverage BAM files (Supplementary Table 1) from [2] using default parameters.

Swedish Samples, Loschbour and Stuttgart

BAM files with genome-level coverage (Supplementary Table 1) from [8] and [1] were realigned to the human genome as outlined in the methods section. MapDamage rescaling[31] was not performed on data from [1] as these samples had been molecularly treated to remove deaminated cytosines, reducing DNA damage associated errors.

La Braña and Mal'ta (MA1)

FASTQ files from [6] and [5] were aligned to the GRCh37 build of the human genome with the mitochondrial sequence removed. Further processing was carried out as described in the methods section. For MA1, mapDamage rescaling[31] was omitted due to the low deamination rates found in this sample[5] .

Ust'-Ishim

A BAM file containing sequences from the Ust'-Ishim genome[3] was filtered to remove reads with mapping quality of less than 30. This sample had been molecularly treated to remove deaminated cytosines so mapDamage rescaling[31] was not performed.

Kostenki

A BAM file containing sequences from the Kostenki genome was downloaded from [4] and duplicate reads were removed. Sequences had already been filtered to have mapping quality > 30 and the first and last 5 bp of all reads had been soft-clipped to a base quality of zero. As terminal bases had been soft-clipped and the sample had been molecularly treated to remove deaminated cytosines, mapDamage rescaling[31] was not carried out.

Otzi the Tyrolean Iceman

Forward FASTQ files were downloaded from [9] and realigned to the GRCh37 build of the human genome with the mitochondrial sequence removed. Sequences were further processed as described in the methods section however reads were not rescaled using mapDamage as the substitution frequencies were outside the bounds of the posterior predictive distribution intervals set by the mapDamage model[31].

Haak ancient data

Genotypes for Eurasian ancient samples (Supplementary Table 1) which overlapped the Human Origins array[1] were downloaded from [7]. Only samples with at least 200,000 called genotypes were used, in-keeping with the level of data from other samples used in analyses. Genotypes had not been called in the first and last two bases of sequence reads and all samples had been UDG-treated to reduce the influence of deaminated cytosines on analyses[7].

Allentoft ancient data

Genotypes for Bronze and Iron Age ancient samples published in [10] were kindly provided by GeoGenetics, Copenhagen. Only samples with at least 200,000 called genotypes were used (Supplementary Table 1).

Soft-clipping ancient data

Due to the SeqTK step carried out prior to alignment (see methods), Kotias, Satsurblia, and Bichon had the first and last two bases of all reads removed. To mirror this step in published ancient data which had not been trimmed in this way, the first and last 2 bp of all sequences were soft-clipped to a base quality of two before genotypes were called (with the exception of data from [7] and [10] for which genotypes were already called).

Supplementary References

1. Lazaridis, I. *et al.* Ancient human genomes suggest three ancestral populations for present-day Europeans. *Nature* **513,** 409–413 (2014).
2. Gamba, C. *et al.* Genome flux and stasis in a five millennium transect of European prehistory. *Nat. Commun.* **5,** 5257 (2014).
3. Fu, Q. *et al.* Genome sequence of a 45,000-year-old modern human from western Siberia. *Nature* **514,** 445–449 (2014).
4. Seguin-Orlando, A. *et al.* Paleogenomics. Genomic structure in Europeans dating back at least 36,200 years. *Science* **346,** 1113–1118 (2014).
5. Raghavan, M. *et al.* Upper Palaeolithic Siberian genome reveals dual ancestry of Native Americans. *Nature* **505,** 87–91 (2013).
6. Olalde, I. *et al.* Derived immune and ancestral pigmentation alleles in a 7,000-year-old Mesolithic European. *Nature* **507,** 225–228 (2014).
7. Haak, W. *et al.* Massive migration from the steppe was a source for Indo-European languages in Europe. *Nature* **522,** 207–211 (2015).
8. Skoglund, P. *et al.* Genomic Diversity and Admixture Differs for Stone-Age Scandinavian Foragers and Farmers. *Science* **344,** 747–750 (2014).
9. Keller, A. *et al.* New insights into the Tyrolean Iceman’s origin and phenotype as inferred by whole-genome sequencing. *Nat. Commun.* **3,** 698 (2012).
10. Allentoft, M. E. *et al.* Population genomics of Bronze Age Eurasia. *Nature* **522,** 167–172 (2015).
11. Rasmussen, M. *et al.* An Aboriginal Australian genome reveals separate human dispersals into Asia. *Science* **334,** 94–98 (2011).
12. Behar, D. M. *et al.* A ‘Copernican’ reassessment of the human mitochondrial DNA tree from its root. *Am. J. Hum. Genet.* **90,** 675–684 (2012).
13. Jostins, L. *et al.* YFitter: Maximum likelihood assignment of Y chromosome haplogroups from low-coverage sequence data. Preprint at http://arxiv.org/abs/1407.7988 (2014).
14. Canfield, V. A. *et al.* Molecular phylogeography of a human autosomal skin color locus under natural selection. *G3* **3,** 2059–2067 (2013).
15. Meshveliani, T. *et al.* Anthromorphic Figurine from Kotias Klde Cave Bulletin of the

Georgian National Museum. *Bulletin of the Georgian National Museum* **IV (49-B):11-17,** (2013).

16. Liubin, V. Pervye svedeija o mezolite gornogo Kavkaza (Osetija) (First evidence concerning the Mesolithic of the Caucasus Highlands (Ossetia)). In: Gurnia N, editor. U istokov drevnikh kul'tur: epokha mezolita (At the Dawn of the Ancient Cultures: The Mesolithic Period). *Moscow & Leningrad:Nakua: Materialy i issledovanija po arkheologii SSSR* 155–163 (1966).
17. Grigolia, G. K. Centraluri Kolhetis neoliti Paluri (The Neolithic of Central Colchis: Paluri). Tbilisi. *Metsniereba* (1977).
18. Arimura, M., Chataigner, C. & Gasparyan, B. Kmlo 2. An Early Holocene site in Armenia. *Neo-Lithics* **2/2009,**17-19 (2009).
19. Nebieridze, L. Darkvetis mravalpeniani ekhi. (The Darkveti Multilayer Rockshelter). Tbilisi: Metsniereba, (1978).
20. Meshveliani, T. *et al.* Mesolithic Hunters at Kotias Klde, Western Georgia: Preliminary Results. *paleo* **33,** 47–58 (2007).
21. Kalandadze, A. N. & Kalandadze. Archaeological Research of Karstic Caves in Tskaltubo region (in Georgian, with Russian summary). *Caves of Georgia* 116–136 (1978).
22. Kalandadze, K. S., Bugianishvili, T., Ioseliani, N., Jikia, M. & Kalandadze, N. Tskaltubo Expedition. The short reports of the archaeological expedition of 1989-1992. (in Georgian with Russian summary). *Tbil. Math. J.* (2004).
23. Pinhasi, R. *et al.* Satsurblia: new insights of human response and survival across the Last Glacial Maximum in the southern Caucasus. *PLoS One* **9,** e111271 (2014).
24. Morel, P. Une chasse à l'ours brun il y a 12'000 ans: nouvelle découverte à la grotte du Bichon (La Chaux-de-Fonds). *Archéologie suisse* **16,** 110–117 (1993).
25. Chauvière F-X . La grotte du Bichon: un site préhistorique des montagnes neuchâteloises: *Archéologie neuchâteloise*, **42** (2008).
26. Simon, C. & Formicola, V. Anatomie générale de l'homme du Bichon. In: Chauvière F-X, editor. La grotte du Bichon: un site préhistorique des montagnes neuchâteloises: *Archéologie neuchâteloise*, **42** (2008).
27. Holt, B. & Churchill, S. Anatomie fonctionnelle du squelette post-crânien de l'homme du Bichon. In: Chauvière F-X, editor. La grotte du Bichon: un site préhistorique des montagnes neuchâteloises: *Archéologie neuchâteloise*, **42** (2008)

28. Iacumin, P. Étude des isotopes stables : détermination du régime alimentaire de l'homme du Bichon. In: Chauvière F-X, editor. La grotte du Bichon: un site préhistorique des montagnes neuchâteloises: *Archéologie neuchâteloise,* **42** (2008).
29. Briggs, A. W. *et al.* Patterns of damage in genomic DNA sequences from a Neandertal. *Proc. Natl. Acad. Sci. U. S. A.* **104,** 14616–14621 (2007).
30. Brotherton, P. *et al.* Novel high-resolution characterization of ancient DNA reveals C > U-type base modification events as the sole cause of post mortem miscoding lesions. *Nucleic Acids Res.* **35,** 5717–5728 (2007).
31. Jónsson, H., Ginolhac, A., Schubert, M., Johnson, P. L. F. & Orlando, L. mapDamage2.0: fast approximate Bayesian estimates of ancient DNA damage parameters. *Bioinformatics* **29,** 1682–1684 (2013).
32. Shapiro, B. & Hofreiter, M. Analysis of ancient human genomes. *Bioessays* **32,** 388–391 (2010).
33. Rohland, N., Siedel, H. & Hofreiter, M. A rapid column-based ancient DNA extraction method for increased sample throughput. *Mol. Ecol. Resour.* **10,** 677–683 (2010).
34. Dabney, J. *et al.* Complete mitochondrial genome sequence of a Middle Pleistocene cave bear reconstructed from ultrashort DNA fragments. *Proc. Natl. Acad. Sci. U. S. A.* **110,** 15758–15763 (2013).
35. Sánchez-Quinto, F. *et al.* Genomic Affinities of Two 7,000-Year-Old Iberian Hunter-Gatherers. *Curr. Biol.* **22,** 1494–1499 (2012).
36. 1000 Genomes Project Consortium *et al.* An integrated map of genetic variation from 1,092 human genomes. *Nature* **491,** 56–65 (2012).
37. Rasmussen, M. *et al.* The genome of a Late Pleistocene human from a Clovis burial site in western Montana. *Nature* **506,** 225–229 (2014).
38. Roostalu, U. *et al.* Origin and Expansion of Haplogroup H, the Dominant Human Mitochondrial DNA Lineage in West Eurasia: The Near Eastern and Caucasian Perspective. *Mol. Biol. Evol.* **24,** 436–448 (2007).
39. Derenko, M. *et al.* Complete mitochondrial DNA diversity in Iranians. *PLoS One* **8,** e80673 (2013).
40. Richards, M. *et al.* Tracing European founder lineages in the Near Eastern mtDNA pool. *Am. J. Hum. Genet.* **67,** 1251–1276 (2000).
41. Vianello, D. *et al.* HAPLOFIND: a new method for high-throughput mtDNA haplogroup

assignment. *Hum. Mutat.* **34,** 1189–1194 (2013).

42. Van Oven, M. & Kayser, M. Updated comprehensive phylogenetic tree of global human mitochondrial DNA variation. *Hum. Mutat.* **30,** E386–94 (2009).
43. Simoni, L., Calafell, F., Pettener, D., Bertranpetit, J. & Barbujani, G. Geographic patterns of mtDNA diversity in Europe. *Am. J. Hum. Genet.* **66,** 262–278 (2000).
44. Brandt, G. *et al.* Ancient DNA reveals key stages in the formation of central European mitochondrial genetic diversity. *Science* **342,** 257–261 (2013).
45. Fernández, E. *et al.* Ancient DNA analysis of 8000 B.C. near eastern farmers supports an early neolithic pioneer maritime colonization of Mainland Europe through Cyprus and the Aegean Islands. *PLoS Genet.* **10,** e1004401 (2014).
46. Bollongino, R. *et al.* 2000 years of parallel societies in Stone Age Central Europe. *Science* **342,** 479–481 (2013).
47. Bramanti, B. *et al.* Genetic discontinuity between local hunter-gatherers and central Europe's first farmers. *Science* **326,** 137–140 (2009).
48. Semino, O. *et al.* Origin, diffusion, and differentiation of Y-chromosome haplogroups E and J: inferences on the neolithization of Europe and later migratory events in the Mediterranean area. *Am. J. Hum. Genet.* **74,** 1023–1034 (2004).
49. Balanovsky, O. *et al.* Parallel evolution of genes and languages in the Caucasus region. *Mol. Biol. Evol.* **28,** 2905–2920 (2011).
50. Cinnioğlu, C. *et al.* Excavating Y-chromosome haplotype strata in Anatolia. *Hum. Genet.* **114,** 127–148 (2004).
51. Jobling, M. A. & Tyler-Smith, C. The human Y chromosome: an evolutionary marker comes of age. *Nat. Rev. Genet.* **4,** 598–612 (2003).
52. Rootsi, S. *et al.* Phylogeography of Y-chromosome haplogroup I reveals distinct domains of prehistoric gene flow in europe. *Am. J. Hum. Genet.* **75,** 128–137 (2004).
53. Semino, O. *et al.* The genetic legacy of Paleolithic Homo sapiens sapiens in extant Europeans: a Y chromosome perspective. *Science* **290,** 1155–1159 (2000).
54. Gronau, I., Hubisz, M. J., Gulko, B., Danko, C. G. & Siepel, A. Bayesian inference of ancient human demography from individual genome sequences. *Nat. Genet.* **43,** 1031–1034 (2011).
55. Prüfer, K. *et al.* The complete genome sequence of a Neanderthal from the Altai Mountains. *Nature* **505,** 43–49 (2013).

56. Li, H. *et al.* The Sequence Alignment/Map format and SAMtools. *Bioinformatics* **25,** 2078–2079 (2009).
57. Green, R. E. *et al.* A Draft Sequence of the Neandertal Genome. *Science* **328,** (2010).
58. Pinhasi, R., Thomas, M. G., Hofreiter, M., Currat, M. & Burger, J. The genetic history of Europeans. *Trends Genet.* **28,** 496–505 (2012).
59. Kong, A. *et al.* Rate of de novo mutations and the importance of father's age to disease risk. *Nature* **488,** 471–475 (2012).
60. Kirin, M. *et al.* Genomic runs of homozygosity record population history and consanguinity. *PLoS One* **5,** e13996 (2010).
61. Pemberton, T. J. *et al.* Genomic patterns of homozygosity in worldwide human populations. *Am. J. Hum. Genet.* **91,** 275–292 (2012).
62. McQuillan, R. *et al.* Runs of homozygosity in European populations. *Am. J. Hum. Genet.* **83,** 359–372 (2008).
63. Reich, D., Thangaraj, K., Patterson, N., Price, A. L. & Singh, L. Reconstructing Indian population history. *Nature* **461,** 489–494 (2009).
64. Sharma, A. N. *Tribal Development in Andaman Islands*. (Sarup & Sons, 2003).
65. Patterson, N. *et al.* Ancient admixture in human history. *Genetics* **192,** 1065–1093 (2012).
66. Hart, K. L. *et al.* Improved eye- and skin-color prediction based on 8 SNPs. *Croat. Med. J.* **54,** 248–256 (2013).
67. Lamason, R. L. *et al.* SLC24A5, a putative cation exchanger, affects pigmentation in zebrafish and humans. *Science* **310,** 1782–1786 (2005).
68. Sturm, R. A. Molecular genetics of human pigmentation diversity. *Hum. Mol. Genet.* **18,** R9–17 (2009).
69. Sturm, R. A. & Duffy, D. L. Human pigmentation genes under environmental selection. *Genome Biol.* **13,** 248 (2012).
70. Basu Mallick, C. *et al.* The light skin allele of SLC24A5 in South Asians and Europeans shares identity by descent. *PLoS Genet.* **9,** e1003912 (2013).
71. Wilde, S. *et al.* Direct evidence for positive selection of skin, hair, and eye pigmentation in Europeans during the last 5,000 y. *Proc. Natl. Acad. Sci. U. S. A.* **111,** 4832–4837 (2014).
72. Relethford, J. H. Hemispheric difference in human skin color. *Am. J. Phys. Anthropol.* **104,** 449–457 (1997).
73. Loomis, W. F. Skin-pigment regulation of vitamin-D biosynthesis in man. *Science* **157,** 501–

506 (1967).

74. Jablonski, N. G. & Chaplin, G. The evolution of human skin coloration. *J. Hum. Evol.* **39,** 57–106 (2000).

75. Walsh, S. *et al.* The HIrisPlex system for simultaneous prediction of hair and eye colour from DNA. *Forensic Sci. Int. Genet.* **7,** 98–115 (2013).

76. Itan, Y., Powell, A., Beaumont, M. A., Burger, J. & Thomas, M. G. The origins of lactase persistence in Europe. *PLoS Comput. Biol.* **5,** e1000491 (2009).
